# Machine-learning to stratify diabetic patients using novel cardiac biomarkers and integrative genomics

**DOI:** 10.1186/s12933-019-0879-0

**Published:** 2019-06-11

**Authors:** Quincy A. Hathaway, Skyler M. Roth, Mark V. Pinti, Daniel C. Sprando, Amina Kunovac, Andrya J. Durr, Chris C. Cook, Garrett K. Fink, Tristen B. Cheuvront, Jasmine H. Grossman, Ghadah A. Aljahli, Andrew D. Taylor, Andrew P. Giromini, Jessica L. Allen, John M. Hollander

**Affiliations:** 1 Division of Exercise Physiology, West Virginia University School of Medicine, PO Box 9227, 1 Medical Center Drive, Morgantown, WV 26505 USA; 2 Mitochondria, Metabolism & Bioenergetics Working Group, West Virginia University School of Medicine, Morgantown, WV 26505 USA; 3 Department of Chemical and Biomedical Engineering, West Virginia University, Morgantown, WV 26505 USA; 4 West Virginia University School of Pharmacy, Morgantown, WV 26505 USA; 5 West Virginia University School of Medicine, Morgantown, WV 26505 USA; 6 Cardiovascular and Thoracic Surgery, West Virginia University School of Medicine, Morgantown, WV 26505 USA

**Keywords:** Epigenetics, Mitochondria, Heart, Machine-learning, CART, SHAP

## Abstract

**Background:**

Diabetes mellitus is a chronic disease that impacts an increasing percentage of people each year. Among its comorbidities, diabetics are two to four times more likely to develop cardiovascular diseases. While HbA1c remains the primary diagnostic for diabetics, its ability to predict long-term, health outcomes across diverse demographics, ethnic groups, and at a personalized level are limited. The purpose of this study was to provide a model for precision medicine through the implementation of machine-learning algorithms using multiple cardiac biomarkers as a means for predicting diabetes mellitus development.

**Methods:**

Right atrial appendages from 50 patients, 30 non-diabetic and 20 type 2 diabetic, were procured from the WVU Ruby Memorial Hospital. Machine-learning was applied to physiological, biochemical, and sequencing data for each patient. Supervised learning implementing SHapley Additive exPlanations (SHAP) allowed binary (no diabetes or type 2 diabetes) and multiple classification (no diabetes, prediabetes, and type 2 diabetes) of the patient cohort with and without the inclusion of HbA1c levels. Findings were validated through Logistic Regression (LR), Linear Discriminant Analysis (LDA), Gaussian Naïve Bayes (NB), Support Vector Machine (SVM), and Classification and Regression Tree (CART) models with tenfold cross validation.

**Results:**

Total nuclear methylation and hydroxymethylation were highly correlated to diabetic status, with nuclear methylation and mitochondrial electron transport chain (ETC) activities achieving superior testing accuracies in the predictive model (~ 84% testing, binary). Mitochondrial DNA SNPs found in the D-Loop region (SNP-73G, -16126C, and -16362C) were highly associated with diabetes mellitus. The CpG island of transcription factor A, mitochondrial (TFAM) revealed CpG24 (chr10:58385262, *P* = 0.003) and CpG29 (chr10:58385324, *P* = 0.001) as markers correlating with diabetic progression. When combining the most predictive factors from each set, total nuclear methylation and CpG24 methylation were the best diagnostic measures in both binary and multiple classification sets.

**Conclusions:**

Using machine-learning, we were able to identify novel as well as the most relevant biomarkers associated with type 2 diabetes mellitus by integrating physiological, biochemical, and sequencing datasets. Ultimately, this approach may be used as a guideline for future investigations into disease pathogenesis and novel biomarker discovery.

**Electronic supplementary material:**

The online version of this article (10.1186/s12933-019-0879-0) contains supplementary material, which is available to authorized users.

## Background

A disconnect continues to persist in the diagnosis and pathogenesis of diabetes-induced cardiovascular dysfunction. While diabetics are at a two to fourfold greater risk of developing cardiovascular diseases [1, 2], understanding how the numerous biochemical markers involved in the pathology integrate and influence disease progression has not been fully explicated. In a clinical setting, the ability to better calculate prognostics of a patient’s health through the integration of biomarkers facilitates the potential for developing personalized and generalized medicine, as well as treatment strategies [3]. While glycated hemoglobin (HbA1c) remains a hallmark for disease diagnosis [4], other biomarkers may exist that more unequivocally define the severity of the pathology, characterize the mechanisms involved, and/or provide a better predictive tool of future cardiovascular events.

Diabetes mellitus is a multifaceted disease, consisting of systemic comorbidities which necessitate a variety of treatment modalities and stratify those affected with the disease [5]. Before the implementation of machine-learning algorithms in medicine, linear statistical models have highlighted measures, such as HbA1c, as diagnostic staples for the evaluation of diabetes mellitus onset and progression [6]. By exploring these previously published metadata sets, machine-learning has been applied in refining the accuracy of biomarkers used to characterize the pathology as well as to highlight vulnerable populations in need of clinical intervention [7]. Machine-learning has also revealed that coupling HbA1c with additional biomarkers, such as 8-hydroxy-2-deoxyguanosine (8-OhdG) and other metabolites, can increase the accuracy of the predictive model and better characterize the severity of the disease [8].

In cardiology, machine-learning approaches have been applied primarily to imaging-based diagnostics, including echocardiography and computed tomography angiography to evaluate cardiovascular health and outcomes [9, 10]. It is estimated that machine-learning applications in the field of cardiovascular research will continue to grow at an exponential rate [11]. While image-derived deep learning models are increasing in popularity, little is known about the predicative power of machine-learning models on basic genomic, epigenomic, proteomic, and metabolomic profiles of the heart. While the beginning of the “big data” age was characterized by the accumulation and compartmentalization of datasets, the recent advent of combining metadata, deep sequencing, and “omics”-based approaches warrants the union between hierarchical predictive algorithms and biological processes. As more patients have access to their personal “omics” profiles, machine-learning will be instrumental in providing feedback for the individual patient and the general population of patients impacted by the disease, enhancing health practice by the caregiver.

While demographic information and physical examination data are more easily procured from patients, the genomic and transcriptomic characterization of a tissue or cell type provides a much finer granularity and uniqueness when predicting phenotypic outcomes in patients [12]. HbA1c, fasting blood glucose, and BMI are examples of easily accessible, valuable measurements when evaluating diabetes progression and onset [6, 13], but genetic components, including epigenetic, epitranscriptomic, single nucleotide polymorphisms (SNPs), and others, provide a wealth of undiscovered information for disease classification. This genetic component may be specifically important when understanding the pathogenesis of diabetes in ethnic groups, when BMI [14, 15] and HbA1c [16] show distinct differences between ethnicities. Though applying patient-matched, genomic information is currently unrealistic for disease diagnosis, it may hold the key for revealing commonalities across ethnic and demographic groups when classifying diabetic onset, progression, and severity.

In the current study, machine-learning was used as a predictive tool to integrate cardiac physiological, biochemical, genomic, and epigenomic biomarker data in a patient-matched fashion and enable determination of type 2 diabetic status. In 50 patients, machine-learning algorithms revealed the interconnectedness between diabetic classification, mitochondrial function, and methylation status. Our study highlights how novel biomarkers can be used to augment existing diagnostic standards as well as provide new, and more precise, methods for identifying the development and severity of type 2 diabetes mellitus in potentially at-risk populations, such as those with prediabetes. While we examine physiological, biochemical, and molecular datasets using machine-learning algorithms, our goal was to understand which features possessed the best predictive accuracies and if these specific features could be used alone, or in conjunction, with HbA1c. The purpose for the inclusion of models that do not rise above 50% predictive accuracy was to contrast them against those models that do rise above 50% in the absence of HbA1c, to determine which biomarkers are the best overall predictors.

## Research design and methods

### Study approval

All tissue and patient information was acquired in a double de-identified fashion, and was approved by the West Virginia University Institutional Review Board and Institutional Biosafety Committee [17]. Patients were all consented by the Heart and Vascular Institute, J.W. Ruby Memorial Hospital at the West Virginia University School of Medicine. Right atrial appendages were removed during open-heart and/or valvular surgeries. Both groups of patients (non-diabetic and type 2 diabetic) who were receiving open-heart surgery could elect for their tissues to be used for research purposes, with no direct or indirect incentivisation. A total of 50 patients were selected for the study (between August 2016 and May 2018), 30 of which were non-diabetic (ND) and 20 that were type 2 diabetic (T2DM) and existed along a spectrum of measured HbA1c levels. Patient inclusion into the study was determined by (a) election for open heart surgery and release of tissue for research purposes (b) was not undergoing surgery due to heart failure, and (c) demographic data was provided. Patient characteristics are provided in Table 1, listing patients classified as non-diabetic and those who have been clinically diagnosed as type 2 diabetic. Patient information is also provided for separation of the groups into those with no diabetes, prediabetes, and type 2 diabetes (Additional file 1: Table S1). Additional file 1: Table S1 contains the matching 50 patient cohort analyzed in Table 1 but with the creation of a new non-diabetic cohort (n = 16), comprised of those individuals with an HbA1c < 5.7, and prediabetic cohort (n = 14), comprised of clinically non-diabetic patients whose HbA1c is between 5.7 and 6.4.

**Table 1:** Patient characteristics and demographic information

Parameter	Non-diabetic	Type 2 diabetic
Age	61.97 ± 2.449	61.16 ± 3.047
Sex	Male = 26, female = 4	Male = 15, female = 5
BMI (kg/m^2^)	29.13 ± 1.08	29.14 ± 1.448
Coronary artery disease	73.33% ± 8.212%	100% ± 0.0%*
Hypertension	86.67% ± 6.312%	94.74% ± 5.263%
Valvular disease	26.67% ± 8.212%	15.79% ± 8.595%
HbA1c	5.567 ± 0.07898	8.016 ± 0.4024*

Groups are considered significantly different if *P* ≤ 0.05 = * compared to non-diabetic. All data are presented as the mean ± standard error of the mean (SEM)

HbA1c: glycated hemoglobin

### Mitochondrial isolation

Mitochondria were isolated from a portion of the right atrial appendage as previously described [18], with modifications by our laboratory [19–21]. Mitochondrial subpopulations of subsarcolemmal and interfibrillar mitochondria were extracted and combined to achieve a total mitochondrial population.

### Electron transport chain (ETC) complex activities

A portion of the tissue from all 50 patients was homogenized using a Polytron PowerGen 500 S1 tissue homogenizer (Fisher Scientific, Hampton, NH) in NP-40 buffer (150 mM NaCl, 50 mM, pH 8.0 Tris-Cl, and 1.0% NP-40). Protein homogenates were used to measure electron transport chain complexes I, III, IV, and V (ATP synthase) spectrophotometrically, as previously described [22] and implemented by our laboratory [19, 23–26]. Protein concentrations were normalized using the Bradford method, with standardization to bovine serum albumin [27].

### Citrate synthase activity

Isolated mitochondria from all 50 patients, was used to measure citrate synthase activity using a colorimetric Citrate Synthase Assay Kit (Sciencell, San Diego, CA), as previously described [28]. Citrate synthase activity, normalized to protein content, was used to determine mitochondrial content.

### Methyltransferase

Using a colorimetric Methyltransferase Assay Kit (Caymen, Ann Arbor, Michigan), *S*-adenosylmethionine (SAM)-dependent methyltransferase activity was assessed, per manufacturer’s instructions. Briefly, nuclear homogenates were used to assess total SAM-dependent methyltransferase activity in all 50 patients.

### DNA 5mC and 5hmC quantification

Using a DNeasy Blood & Tissue Kit (Qiagen, Hilden, Germany), DNA was isolated from both 10 mg of atrial appendage tissue and mitochondria, per manufacturer’s instructions. Levels of 5-methylcytosine (5mC) and 5-hydroxymethylcytosine (5hmC) were measured through a 5mC and 5hmC ELISA Kit (Zymo Research, Irvine, CA), per manufacturer’s instructions. DNA was quantified using a NanoDrop™ 1000 Spectrophotometer (Thermo Fisher, Waltham, MA). 100 ng of nuclear (tissue extract) and mitochondrial DNA were used to assess total 5mC and 5hmC content spectrophotometrically for all 50 patients.

### Western blotting

Using 4–12% gradient gels, immunoblotting was performed through MES SDS-PAGE, as previously described [21, 26, 29–31]. Protein was normalized using the Bradford method. Primary antibodies used in the study included: anti-TFAM, transcription factor A, mitochondrial, 1:500 (SCBT, Dallas, TX), anti-GAPDH 1:1000 (Abcam, Cambridge, MA). The secondary antibody used in the study was a goat anti-mouse IgG (H&L) horseradish peroxidase (HRP) conjugate 1:10,000 (Thermo Fisher). GAPDH expression was used to normalize protein content. Chemiluminescence was measured through Radiance Chemiluminescent Substrate (Azure Biosystems, Dublin, CA), per manufacturer’s instructions and imaged using the G:Box Bioimaging system (Syngene, Frederick, MD). Images were captured through GeneSnap/GeneTools software (Syngene). Densitometry was analyzed using ImageJ and Fiji Software (NIH, Bethesda, MD). Data is represented as optical density with arbitrary units.

### Chromatin immunoprecipitation (ChIP)-qPCR

The SimpleChIP^®^ Plus Sonication Chromatin IP Kit (Cell Signaling Technology, Danvers, MA) was used, per manufacturer’s instructions. Briefly, 100 mg of atrial tissue was minced into ~ 2 mm^3^ pieces and treated with freshly prepared 37% formaldehyde for 30 min. Sonicated DNA was assessed for sheering and further immunoprecipitated with anti-TFAM (SCBT) bound Protein G magnetic beads overnight at 4 °C. The beads were washed, DNA reverse cross-linked, and DNA eluted as previously described [24, 32]. DNA bound to TFAM was further examined through qPCR. 2% chromatin inputs for each sample were used for normalization of expression. An Applied Biosystems 7900HT Fast Real-Time PCR system (Applied Biosystems, Foster City, CA) was used to assess expression through SYBR Green. Quantification was achieved using the 2^−ΔΔCT^ method [33]. All primer pairs to assess the mitochondrial D-Loop are provided (Additional file 1: Table S2).

### Overhang-bisulfite sequencing

DNA was extracted from patient tissue as described above. DNA was bisulfite-treated using the EZ DNA Methylation-Lightning Kit (Zymo Research), per manufacturer’s instructions. Primers were designed for the CpG island of TFAM; primer set 1 amplified the 3′ end and primer set 2 amplified the 5′ end of the CpG island (Additional file 1: Table S2). Bisulfite DNA was prepared for sequencing using a 2-Step PCR amplification process [34]. Step-1 PCR adapters included a base pairing region (~ 23 bp) and an overhang Illumina adapter arm (~ 33 bp). Bisulfite DNA was PCR amplified using Step-1 primers utilizing Platinum™ Taq DNA Polymerase (Thermo Fisher), run on 2% agarose gels, extracted through a QIAquick Gel Extraction Kit (Qiagen), and DNA purified. DNA was then further amplified using Step-2 Illumina barcoded adapters and prepared using a 300-cycle MiSeq Reagent Micro Kit v2 (Illumina, San Diego, CA). PCR amplicons were sequenced on the MiSeq with paired-end (PE) 250 base pair reads. Files were aligned to the bisulfite converted reference genome GRCh38 release 94 implementing Bismark [35, 36]. Alignment was obtained through Bismark using the Bowtie2 [37] engine using “non-directional” and “paired-end.” Complete sequencing code is provided (https://github.com/qahathaway/WVU_Machine-Learning-50/tree/master).

### Mitochondrial SNP sequencing

Mitochondrial DNA was isolated as described above and further amplified using the REPLI-g Mitochondrial DNA Kit (Qiagen), per manufacturer’s instructions. Libraries for amplified DNA were prepared using the MiSeq Reagent Kit v3 (Illumina). Mitochondrial DNA was sequenced on the MiSeq with paired-end (PE) 300 base pair reads. Files were aligned to the reference genome GRCh38 release 94 through Bowtie2 using “sensitive-local.” BAM files were sorted, run through variant calling, and single-nucleotide polymorphisms (SNPs) were identified in the mitochondria through SAMtools [38–40]. Complete sequencing code is provided (https://github.com/qahathaway/WVU_Machine-Learning-50/tree/master).

### Machine-learning algorithms

**Fig. 1:**
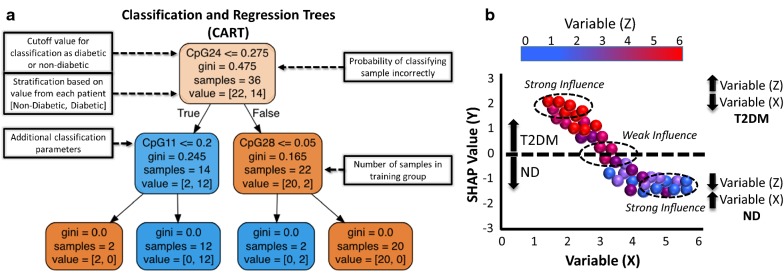
Overview of machine-learning using Classification and Regression Trees (CART) and SHapley Additive exPlanations (SHAP). **a** Classification trees begin with a specific parameter that most successfully partitions the samples, such as CpG24 methylation, and determine the probability of correctly delineating a population into classifications, such as non-diabetic and diabetic, through a discrete value of the parameter (e.g. 0.275). The delineation is then given a probability score (i.e. 0.475, or a 47.5% chance of classifying the sample incorrectly), assigned a label, and further passed on to other parameters in the tree (e.g. CpG11 methylation and CpG28 methylation). As the samples progress through the tiers of the tree, the Gini impurity gets smaller, more accurately delineating samples that make it to that particular “truth” statement. **b** An example of how SHAP illustrates sample distribution. The “SHAP Value” delineates between a condition being true (value > 0.0, T2DM) and it being false (value < 0.0, ND). The more a specific value of a sample influences the composition of the model, the farther the point will migrate away from zero on the y-axis. If the value of a sample does not influence the model, it will reside near or at zero on the y-axis. In the example, a larger value of “X” and lower value of “Z” are highly predictive of the patient being ND, with these values strongly influencing the model “Y”. CpG: cytosine nucleotide followed by a guanine nucleotide; ND: non-diabetic; T2DM: type 2 diabetic

Decision tree classifier algorithms were created in python (v3.6.5) using *scikit*-*learn* [41] and *pandas* [42] libraries (Fig. 1a). In binary classification, patient labels were determined through previous clinical diagnoses, where diabetic status was retained even if current HbA1c levels were below 6.5%. In multiple classification, patients with an HbA1c value in the range of 5.7% to 6.4% were designated as having prediabetes. Due to this, the HbA1c feature was excluded from all tested trials, and the derived accuracies are in comparison to that of the “perfect” accuracy obtained from HbA1c classification. A decision tree classifier model was created using the functions of *scikit*-*learn tree*. The data file was split into 80% training and 20% testing partitions using a defined seed value. Different seeds were chosen for each set to maintain the training and testing set distributions. Selected seed values maintained a binary classification testing set of five diabetics and five non-diabetics. In the multiple classification testing set, seed values maintained a distribution of three diabetics, three non-diabetics, and four pre-diabetics. Seeds were only chosen such that the resulting training and testing accuracies were similar; ensuring that the created classification tree did not over fit to the small sample size and remained generalizable to future testing samples.

Within the model, tenfold cross validation was implemented. CART analysis was then performed on each of the datasets using the *scikit*-*learn* model, and the features of importance extracted using the *feature importance* parameter. The physiological/biochemical, genomic, and epigenomic datasets were combined into a single file to serve as the “all features” dataset used to extract the best and most influential biomarkers. For each trial, selected combinations of biomarkers from each dataset were utilized, and within each trial CART analysis was performed five times. After each of the five iterations, average feature importance, average training, and average testing accuracies were obtained. Standard deviations were taken over each of the five iterations per trial. For each dataset, the first iteration of CART analysis included all biomarkers of each set. If the average feature importance was less than 1%, the feature was no longer included in subsequent trials. After all iterations, if the average feature importance was less than or equal to 8% it was assigned to a category titled “other.” These same trials were repeated with other default *scikit*-*learn* machine-learning frameworks (Logistic Regression, Linear Discriminant Analysis, K-Nearest Neighbors Classifier, Gaussian Naïve Bayes, and Support Vector Machine). Tenfold cross validation and the same seed parameters were used in analysis of these five models and the resulting training and testing accuracies are provided (Additional file 1: Tables S3–S10). The six models tested include few modifications and utilize no additional regularization techniques aside from those default to the *scikit*-*learn* library models. Only the Support Vector Machine model received modifications, with the probability parameter set to “true” to provide probability estimates for each data point and a linear kernel used over the default Radial Basis Function ‘rbf’ kernel. Code for analyses is also provided (https://github.com/qahathaway/WVU_Machine-Learning-50/tree/master).

### Machine-learning feature extraction and accuracy

To determine which model(s) would provide the most accurate predictions on the current data, we assessed the 345 total features across all six models in binary (Table 2) and multiple (Table 3) classification of diabetic status. Through assessment of individual datasets (i.e. physiological/biochemical, genetic, and epigenetic), a set of 18 features was further used to classify diabetic status in binary (Additional file 1: Table S11) and multiple (Additional file 1: Table S12) classification. Model analysis was enacted for each dataset, and the established tenfold cross validation and seed parameters for binary and multiple classification were utilized. Each dataset was tested five times per model. Averages were obtained for training accuracy, training standard deviation, testing accuracy, testing standard deviation, model average F1 score, and area under the curve (AUC). AUC values were provided for each of the six tested algorithms for the testing data under binary classification using the *roc_auc_score* function of *scikit*-*learn*, but not for multiple classification. AUC was determined as 1.0 for LR and SVM models when evaluating the 345 total features due to the large sample size of biomarkers. From the available 345 features, two predictors were chosen that perfectly distinguished diabetic and nondiabetic status for this particular dataset. As such, these AUC values were removed from Table 2 for LR and SVM, as this was not an accurate indicator of the model’s predictive ability. As the feature set was restricted to the 18 “best” features from each dataset, AUC values of 1.0 were no longer observed (Additional file 1: Tables S11 and S12).

**Table 2:** Overview of 6 machine-learning model analysis on all 345 features in binary classification

Model	Training	Training (StDev)	Testing	Testing (StDev)	F1 score	Important features	Important feature bias	AUC
LR	0.608	0.301	0.667	0.0	0.640	Complex III, Complex I, CpG31, CpG28, CpG30, Complex IV, CpG8, CpG4, CpG12, Age	(− 2.688), (− 1.688), (1.648), (− 1.163), (− 1.016), (0.982), (0.945), (0.887), (0.882), (0.848)	NA
LDA	0.567	0.203	0.556	0.0	0.400	SNP16245, SNP16344, SNP151, SNP5463, SNP4295, SNP13722, SNP94, SNP15884, SNP9055, SNP477	(− 3.896E+15), (− 3.896E+15), (− 3.896E+15), (− 3.896E+15), (− 2.719E+15), (− 2.719E+15), (3.398E+14), (3.398E+14), (3.398E+14), 0.266	0.700
KNN	0.642	0.239	0.444	0.0	0.430	NA	NA	0.600
NB	0.725	0.227	0.778	0.0	0.780	Mito 5hmC, Methyltransferase	(1.000), (0.000)	0.775
SVM	0.583	0.337	0.667	0.0	0.640	Complex III, CpG31, Complex I, CpG28, CpG8, CpG22, CpG12, CpG29, CpG4, CpG35	(− 0.732), (0.488), (− 0.443), (− 0.372), (0.350), (− 0.349), (0.322), (− 0.260), (0.259), (0.257)	NA
CART	0.790	0.209	0.711	0.1	0.714	CpG 24, CpG 28, Nuc 5mC, CpG11, CpG23, CpG1, CpG4	(0.587%), (0.213%), (0.040%), (0.040%), (0.040%), (0.040%), (0.040%)	0.715

Model analysis was conducted five times and averages are reported for the resulting training accuracy, training standard deviation, testing accuracy, testing standard deviation, F1 score, and *area under the curve* (AUC). Important biomarker features associated with each trained model are provided along with the associated influence value for each feature. Important features are listed in order of influence within the model. LR, LDA, SVM feature bias exists as an influence parameter where magnitude dictates feature influence. A positive influence value indicates the biomarker favors classification towards one label while a negative value indicates favorable classification of the opposite label. The larger the magnitude, the more strongly that feature shifts classification. NB feature influence indicates the most important biomarker per class in binary (0,1) classification schemes. CART feature bias percentages indicate feature influence on the created classification tree. Larger percentages indicate a feature that arises near the beginning of a tree before subsequent branching. Influence is not provided for KNN due to model restrictions

**Table 3:** Overview of 6 machine-learning model analysis on all 345 features in multiple classification

Model	Training	Training (StDev)	Testing	Testing (StDev)	F1 score	Important features	Important feature bias
LR	0.333	0.207	0.444	0.0	0.430	Complex V, CpG35, BMI, CpG38, CpG18, CpG40, CpG19, CpG23, Complex IV, CpG25	(− 2.417), (− 2.214), (1.942), (− 1.541), (− 1.313), (− 0.994), (− 0.881), (− 0.824), (− 0.812), (0.8071)
LDA	0.433	0.178	0.333	0.0	0.170	SNP11167, SNP10506, SNP16309, SNP16343, SNP2294, SNP14139, SNP16162, SNP3672, SNP8642, SNP143	(− 4.623E+14), (− 4.623E+14), (− 4.623E+14), (− 4.623E+14), (− 4.623E+14), (− 4.623E+14), (− 4.623E+14), (− 4.623E+14), (− 4.623E+14), (5.779E+13)
KNN	0.358	0.239	0.444	0.0	0.450	NA	NA
NB	0.425	0.243	0.778	0.0	0.780	Methyltransferase, Mito 5hmC, Nuc 5 hmC	(0.000), (1.000), (2.000)
SVM	0.442	0.163	0.556	0.0	0.520	Complex V, BMI, Complex III, Complex I, Complex IV, CpG31, Age, CpG19, CpG22, CpG6	(− 0.943), (0.754), (0.561), (− 0.383), (− 0.344), (0.307), (− 0.287), (− 0.268), (− 0.210), (0.198)
CART	0.660	0.257	0.556	0.0	0.558	CpG24, TFAM CpG, TFAM Non-CpG, BMI, SNP94, Complex IV, SNP8557, CpG7, SNP242, SNP13722, Complex III, Mito 5mC	(0.328%), (0.206%), (0.176%), (0.137%), (0.016%), (0.045%), (0.016%), (0.016%), (0.016%), (0.016%), (0.016%), (0.016%)

Model analysis was conducted five times and averages are reported for the resulting training accuracy, training standard deviation, testing accuracy, testing standard deviation, and F1 score. Important biomarker features associated with each trained model are provided along with the associated influence value for each feature. Important features are listed in order of influence within the model. LR, LDA, SVM feature bias exists as an influence parameter where magnitude dictates feature influence. A positive influence value indicates the biomarker favors classification towards one label while a negative value indicates favorable classification of the opposite label. The larger the magnitude, the more strongly that feature shifts classification. NB feature influence indicates the most important biomarker per class in multiple (0,1,2) classification schemes. CART feature bias percentages indicate feature influence on the created classification tree. Larger percentages indicate a feature that arises near the beginning of a tree before subsequent branching. Influence is not provided for KNN due to model restrictions

Extracted important features and corresponding feature bias within each model, with the exception of KNN, were determined and are provided for binary and multiple classification. CART feature importance was extracted from the trained model using the *feature importance* parameter. For the NB model, feature influence was determined using the *predict_log_proba* function to return the most important biomarker per class in both binary (0,1) and multiple (0,1,2) classification schemes. Feature importance was not determined for the KNN model due to the restrictions of the default model. LDA, LR, and SVM feature influence was determined by taking the magnitude of the model coefficients, *coef_* parameter, times the standard deviation of that biomarker in the testing data. The resulting values are ranked based off magnitude and are reported with sign under the “Important Feature Bias” (Tables 2 and 3, Additional file 1: Tables S11 and S12). A positive influence value indicates a biomarker favoring classification towards one label while a negative value favors the opposite classification label. The larger the magnitude, the more strongly that feature shifts classification.

### SHapley Additive exPlanations (SHAP)

SHAP framework, from slundberg (https://github.com/slundberg/shap), was used to visually explain the classification trees developed for the 50 patient samples using an XGBoost model (Fig. 1b) [43–45]. Figure 1b illustrates how SHAP plots are presented in three dimensions, with the “X” and “Y” dimensions plotted spatially while the “Z” dimension is indicated only through color; allowing for the examination of how variables, “X” and “Z”, can influence the nature of the model and classification of patients, “Y”, into non-diabetic and diabetic cohorts. Through Jupyter Notebook (v0.34.9) [46] with a Python 3 kernel and importing *pandas*, *shap*, and *xgboost* libraries, SHAP plots were derived. Delineation of binary and multiple classification systems are defined above. The entire 50 patient population was utilized during training of the XGBoost model and both the patient biomarker values and patient labels were provided during training. The XGBoost and SHAP tree explainer utilized were left unaltered. The number of influential features shown in the summary plot were selected using the *max display* parameter. Plot generation utilized *force plot*, *dependency plot*, and *summary plot* SHAP functions. Force plots depict the effect of biomarker values on the model’s output. Dependency plots relate specific biomarker values to model prediction and show how the chosen biomarker depends on other tested biomarkers. Summary plots depict the top influential biomarkers and how they influence the model prediction. Code for analyses is also provided (https://github.com/qahathaway/WVU_Machine-Learning-50/tree/master).

### Statistics

Significance was determined using a two-tailed Student’s t-test or one-way analysis of variance (ANOVA), where appropriate. Tukey’s multiple comparisons test was implemented following the ANOVA to derive significance between multiple groups. Differences between groups were considered statistically different if *P* ≤ 0.05, denoted by * if statistically different from non-diabetic or # if statistically different from pre-diabetic. All data are presented as the mean ± standard error of the mean (SEM).

### Data availability

Mitochondrial DNA-Seq: Sequence Read Archive PRJNA520920 https://dataview.ncbi.nlm.nih.gov/?search=SUB5124294.

TFAM Promoter Methylation Amplicon-Seq: Sequence Read Archive PRJNA520920 https://dataview.ncbi.nlm.nih.gov/?search=SUB5125264.

Bioinformatics and Machine-learning Scripts: Github https://github.com/qahathaway/WVU_Machine-Learning-50/tree/master.

## Results

Alterations to the interaction networks that exist between the nucleus and mitochondrion play a significant role in the development of diabetic cardiomyopathy [47–50]. As a result, we wanted to determine how observed changes in these parameters could predict diabetic status using machine-learning algorithms. All of the machine-learning algorithms in this study implemented to draw conclusions were constructed around tree ensembles, such as Classification and Regression Trees (CART). CART algorithms proved to have the overall highest testing and training accuracies when compared to other models (Additional file 1: Tables S3–S10), while also performing superiorly in multiple classification of prediabetes (Tables 2, 3). When examining the testing, training, and area under the curve (AUC) values that depict model performance, CART performed consistently at, or near, the top of the six models in both the binary (Table 2) and multiple (Table 3) classification sets when assessing all 345 features. SHapley Additive exPlanations (SHAP) which implement CART functions were used to provide binary (non-diabetic or type 2 diabetic) as well as multiple (non-diabetic, prediabetic, and type 2 diabetic) classification analyses. SHAP analysis maps a particular biomarker’s numeric values to a computationally defined SHAP value that represents the degree to which specific biomarker values classify the patient to a particular label (non-diabetic or type 2 diabetic). We wanted to demonstrate how machine-learning algorithms, applied across a variety of health outcome datasets, could be implemented to identify novel biomarkers, with and without HbA1c, to provide better assessment of type 2 diabetes mellitus. By presenting each dataset distinctly, we were able to assess which biomarkers provided the best overall predictive power.

### Physiological and biochemical analyses

**Fig. 2:**
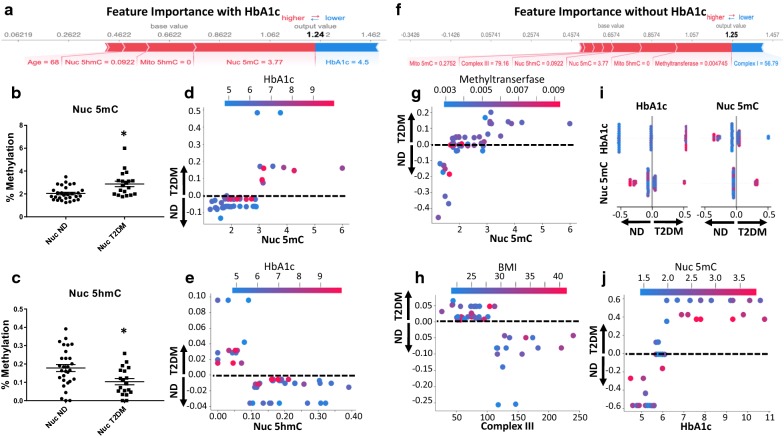
Feature importance of physiological and biochemical characteristics from patients. **a** Using HbA1c for binary classification representing the factors positively (red) and negatively (blue) impacting the construction of the model, with size of the bars depicting importance. The **b** total nuclear methylation and **c** total nuclear hydroxymethylation of patients. SHAP binary depiction of the interaction between **d** total nuclear methylation and **e** total nuclear hydroxymethylation and HbA1c levels. **f** Not including HbA1c for binary classification representing the factors positively (red) and negatively (blue) impacting the construction of the model, with size of the bars depicting importance. SHAP binary depiction without HbA1c of the interaction between **g** total nuclear methylation and methyltransferase activity and **h** electron transport chain complex III and BMI. Examining the multiple classification effects of prediabetes, **i** A modified T-Plot where the main effects of biomarkers on the prediction output are shown along the diagonal axis whereas interaction effects are shown off the diagonal. SHAP depiction of patient separation with the individual and correlated effects of HbA1c and total nuclear methylation. SHAP multiple classification depiction of the interaction between **j** total nuclear methylation and HbA1c. SHAP values > 0.0 are diabetic (T2DM), SHAP values < 0.0 are non-diabetic (ND), SHAP values = 0 are either ND or T2DM without influence on the model. Groups are considered significantly different if *P* ≤ 0.05 = * compared to non-diabetic. All data are presented as the mean ± standard error of the mean (SEM). ND: non-diabetic; T2DM: type 2 diabetic; Nuc: nuclear; Mito: mitochondrial; 5mC: 5-methylcytosine; 5hmC: 5-hydroxymethylcytosine; HbA1c: glycated hemoglobin; binary: no diabetes and diabetes; multiple: no diabetes, prediabetes, and type 2 diabetes

Those with type 2 diabetes mellitus had significantly lower electron transport chain (ETC) complex I and III activities, along with a decreased methyltransferase activity (Additional file 1: Table S13). Using CART analysis and machine-learning, total nuclear methylation, total mitochondrial hydroxymethylation, and total nuclear hydroxymethylation were shown to be the most important factors influencing the model (Fig. 2a). Total nuclear methylation was also shown to be significantly increased in type 2 diabetics (Fig. 2b) with a corresponding decrease in total nuclear hydroxymethylation (Fig. 2c). Nuclear methylation increased as HbA1c levels increased (Fig. 2d) while the rate of hydroxymethylation, generally inversely correlated with methylation levels, decreased as HbA1c increased (Fig. 2e). Methyltransferase activity, total mitochondrial hydroxymethylation, total nuclear methylation, and total nuclear hydroxymethylation were shown to be important features in predicting type 2 diabetes mellitus in the absence of HbA1c (Fig. 2f). High *s*-adenosyl methionine (SAM) methyltransferase activity was also shown to be strongly associated with lower total nuclear methylation levels in the absence of HbA1c (Fig. 2g).

A decrease in mitochondrial ETC complex III activity was associated with a higher BMI (Fig. 2h). While those who were considered to be prediabetic (HbA1c 5.7–6.4) did not show significant differences between any of the biochemical measures except total TFAM CpG methylation (Additional file 1: Table S14), total nuclear methylation was still shown to provide partial classification of patients into non-diabetic, prediabetic, and type 2 diabetic designations (Fig. 2i, j). CART tenfold cross validation confirmed findings for binary [testing (0.838), training (0.7448)] and multiple [testing (0.598), training (0.545)] classification (Additional file 1: Figure S1A–D).

### Genomic analyses

**Fig. 3:**
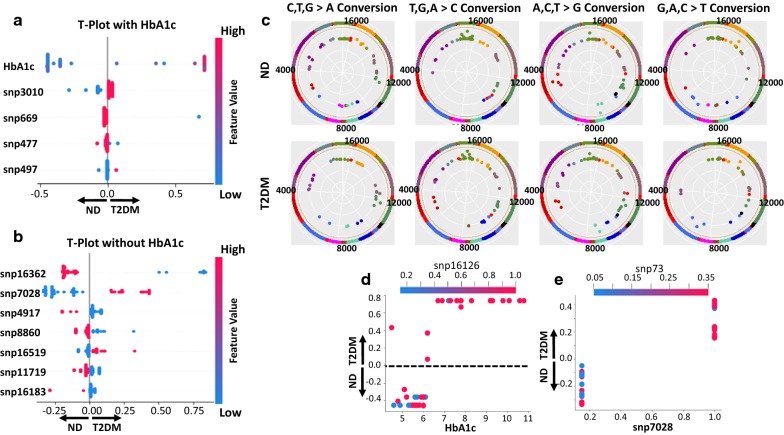
Feature importance of mitochondrial DNA SNPs from patients. **a** The most important predictive parameters using binary classification with HbA1c, the absolute value of a feature being high (red) or low (blue) depicting diabetic (right-side) or non-diabetic (left-side) status. **b** The most important predictive parameters using binary classification without HbA1c, the absolute value of a feature being high (red) or low (blue) depicting diabetic (right-side) or non-diabetic (left-side) status. **c** Frequency of mitochondrial DNA SNPs by nucleotide converted in ND and T2DM patients; increasing frequency of SNPs occurring in the patient population are depicted by movement closer to the mitochondrial DNA strand. **d** SHAP binary depiction with HbA1c of the interaction between SNP16126 and HbA1c. **e** SHAP binary depiction without HbA1c of the interaction between SNP7028 and SNP73. SHAP values > 0.0 are diabetic (T2DM), SHAP values < 0.0 are non-diabetic (ND), SHAP values = 0 are either ND or T2DM without influence on the model. ND: non-diabetic; T2DM: type 2 diabetic; HbA1c: glycated hemoglobin; binary: no diabetes and diabetes; multiple: no diabetes, prediabetes, and type 2 diabetes

The complete mitochondrial genomes of all patients were sequenced, and a list of all single nucleotide polymorphisms (SNPs) was compiled. The binary nature of SNPs, i.e. either being converted or not, allowed the dynamic HbA1c levels to influence the machine-learning model much more efficiently (Fig. 3a). When HbA1c was removed, classification of diabetic or non-diabetic through SNPs was much more apparent, revealing that the 16,362 base pair was most significantly impacted (Fig. 3b). When examining the distribution of SNPs across the mitochondrial genome, the most significant area for base pair alterations to occur was shown to be the D-Loop, or control region (Fig. 3c).

The D-Loop (mtDNA 16,025–576 bp), as compared to all other regions in the mitochondrial genome, contained the highest frequency of SNPs used to predict type 2 diabetes mellitus (Fig. 3a, b, Additional file 1: Figure S2A, B). We further investigated how transcription factor binding could be altered at the D-Loop through chromatin immunoprecipitation (ChIP) of mitochondrial transcription factor A, mitochondrial (TFAM). Though protein levels of TFAM were unchanged (Additional file 1: Figure S3A), ChIP-qPCR revealed decreased binding of TFAM to the proximal and distal end of the control region in type 2 diabetics (Additional file 1: Figure S3B). SNPs near the replication of the H strand (Fig. 3d) or at the end of the D-Loop region (Fig. 3e) could impact TFAM binding and mitochondrial genome transcription. CART tenfold cross validation confirmed findings for binary [testing (0.79), training (0.92)] and multiple [testing (0.576), training (0.808)] classification (Additional file 1: Figure S2A–D).

### Epigenomic analyses

**Fig. 4:**
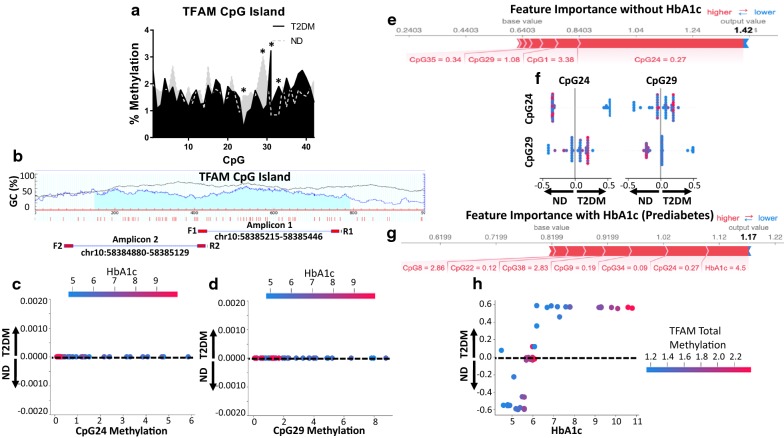
Feature importance of CpG island methylation of TFAM from patients. **a** Methylation across the promoter CpG region of the TFAM gene was determined using overhang bisulfite sequencing. **b** Experimental paradigm for amplification of the bisulfite-converted DNA for 23 CpG sites proximal (Amplicon 1) and 19 CpG sites distal (Amplicon 2) to the TFAM start site. SHAP binary depiction with HbA1c of the interaction between **c** CpG24 methylation and HbA1c and **d** CpG29 methylation and HbA1c. **e** Not including HbA1c for binary classification representing the factors positively (red) and negatively (blue) impacting the construction of the model, with size of the bars depicting importance. **f** A modified T-Plot where the main effects of biomarkers on the prediction output are shown along the diagonal axis whereas interaction effects are shown off the diagonal. SHAP binary depiction without HbA1c of patient separation with the individual and correlated effects of CpG24 methylation and CpG29 methylation. **g** Using HbA1c for multiple classification representing the factors positively (red) and negatively (blue) impacting the construction of the model, with size of the bars depicting importance. **h** SHAP multiple classification depiction with HbA1c of the interaction between TFAM gene total methylation and HbA1c. SHAP values > 0.0 are diabetic (T2DM), SHAP values < 0.0 are non-diabetic (ND), SHAP values = 0 are either ND or T2DM without influence on the model. Groups are considered significantly different if *P* ≤ 0.05 = * compared to non-diabetic. All data are presented as the mean ± standard error of the mean (SEM). ND: non-diabetic; T2DM: type 2 diabetic; HbA1c: glycated hemoglobin; CpG: cytosine nucleotide followed by a guanine nucleotide; TFAM: transcription factor A, mitochondrial; binary: no diabetes and diabetes; multiple: no diabetes, prediabetes, and type 2 diabetes

The cytosine nucleotide followed by a guanine nucleotide (CpG) island of TFAM was examined (Fig. 4a), using overhang bisulfite PCR to amplify regions of the island for sequencing (Fig. 4b). Though total methylation of the gene was low (~ 3%) and showed no significant differences between non-diabetic and type 2 diabetic patients (Additional file 1: Table S13), site-specific CpG island methylation changes revealed significant differences between groups (Fig. 4a). Specifically, the 24th (CpG24) and 29th (CpG29) CpGs in the amplified region revealed significant hypomethylation in type 2 diabetic patients (Fig. 4c, d).

Without using the HbA1c parameter, methylation levels at CpG24, 1, 29, and 35 were shown to be significant contributors to the prediction of diabetic status (Fig. 4e). When comparing the interactions of CpG24 and CpG29, methylation levels at CpG24 were shown to allow distinct separation of the non-diabetic and type 2 diabetic population (Fig. 4f). CpG24 methylation remained a primary predictor, even in the presence of HbA1c for multiple classification (Fig. 4g). Examining total methylation of the TFAM CpG island, prediabetics exhibited an overall increase in methylation, while non-diabetics and type 2 diabetics with similar HbA1c levels showed much lower expression (Fig. 4h). CART tenfold cross validation confirmed findings for binary [testing (0.79), training (0.925)] and multiple [testing (0.668), training (0.767)] classification (Additional file 1: Figure S4A–D).

### Best/combined analyses

**Fig. 5:**
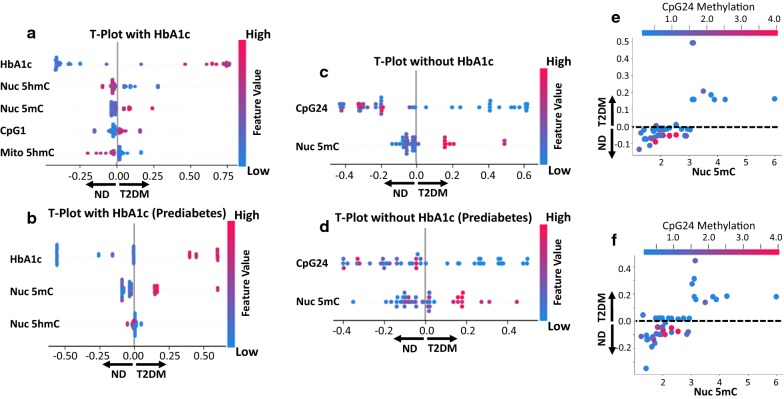
Feature importance of best factors combined from patients. The most important predictive parameters using **a** binary and **b** multiple classification with HbA1c, the absolute value of a feature being high (red) or low (blue) depicting diabetic (right-side) or non-diabetic (left-side) status. The most important predictive parameters using **c** binary and **d** multiple classification without HbA1c, the absolute value of a feature being high (red) or low (blue) depicting diabetic (right-side) or non-diabetic (left-side) status. SHAP **e** binary and **f** multiple classification depiction without HbA1c of the interaction between total nuclear methylation and CpG24 methylation. SHAP values > 0.0 are diabetic (T2DM), SHAP values < 0.0 are non-diabetic (ND), SHAP values = 0 are either ND or T2DM without influence on the model. ND: non-diabetic; T2DM: type 2 diabetic; HbA1c: glycated hemoglobin; CpG: cytosine nucleotide followed by a guanine nucleotide; Nuc: nuclear; 5mC: 5-methylcytosine; binary: no diabetes and diabetes; multiple: no diabetes, prediabetes, and type 2 diabetes

**Fig. 6:**
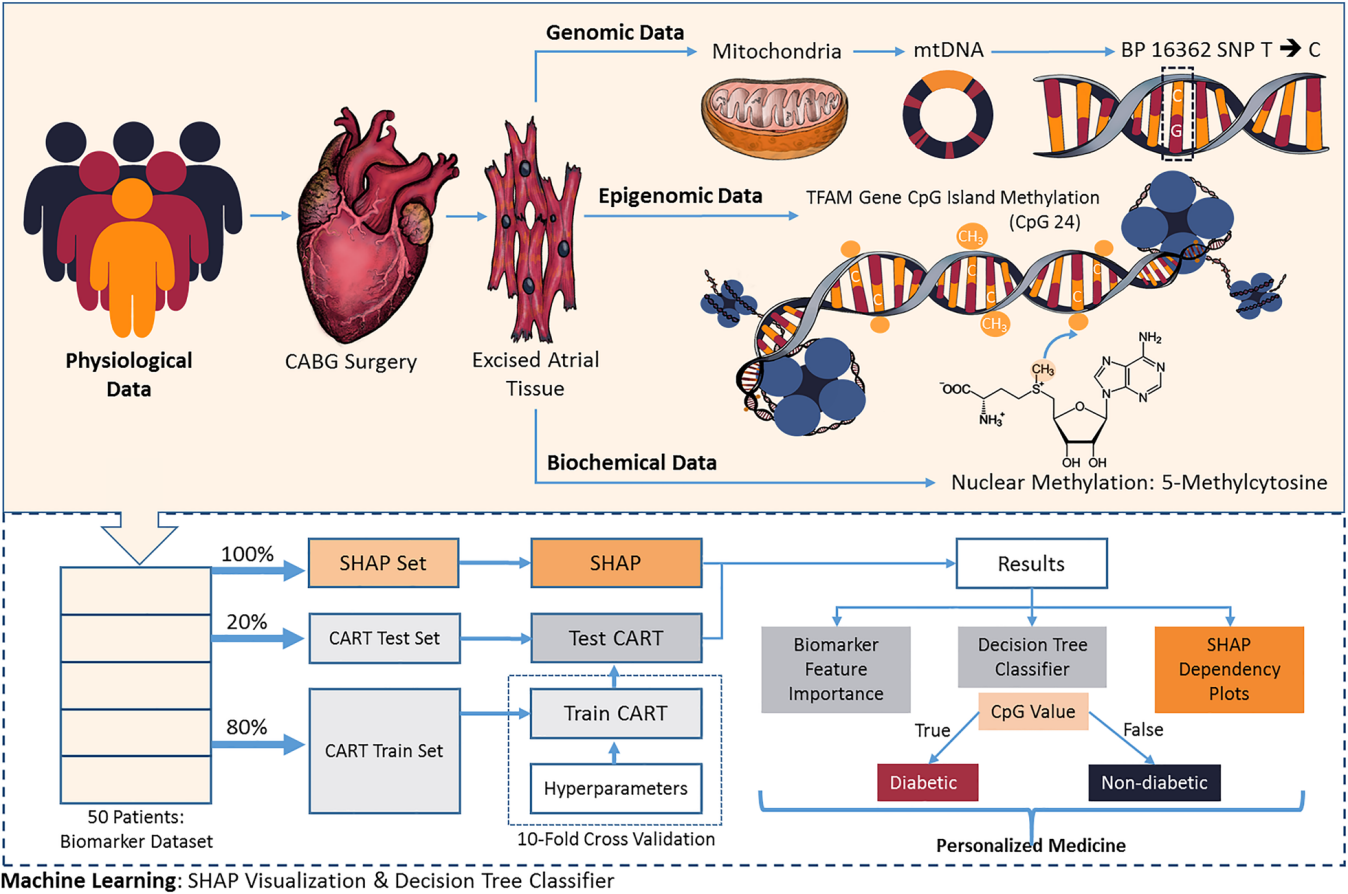
Overview of machine-learning pipeline implementing biological variables across a spectrum of gathered information. From the patient population undergoing coronary artery bypass graft surgery (CABG), physiological parameters (demographics, health reports, etc.) and atrial tissue were used for subsequent analyses. From cardiac tissue genomic (mitochondrial DNA), epigenomic (TFAM promoter CpG methylation), and biochemical (nuclear and mitochondrial function) were assessed. Cumulatively, the biological data was processed through tree ensembles in SHAP and validated through CART analysis with tenfold cross validation. Using these machine-learning algorithms, graphical depictions and biomarker feature importance are able to be derived, allowing for prediction of the onset and progression of diabetes. Ultimately, by using biological data at the genomic and epigenomic level, it allows for precision medicine approaches and more personalized diagnostics and prognostics. TFAM: transcription factor A, mitochondrial; mtDNA: mitochondrial DNA; CpG: cytosine nucleotide followed by a guanine nucleotide; CART: Classification and Regression Trees; SHAP: SHapley Additive exPlanations

Those physiological, biochemical, genomic, and/or epigenomic markers that provided the best association within their class for predicting type 2 diabetes mellitus status were used in the final analyses. With the combined list of features, CART algorithms continued to perform consistently at, or near, the top for testing and training accuracies in binary (Additional file 1: Table S11) and multiple (Additional file 1: Table S12) classification. Total nuclear hydroxymethylation and total nuclear methylation levels provided the most powerful predictors in delineating between binary (non-diabetic and type 2 diabetic) (Fig. 5a) and multiple (non-diabetic, prediabetic, type 2 diabetic) (Fig. 5b) classifications, distinguishing them as potentially suitable biomarkers to accompany diagnostic practices using HbA1c. When using machine-learning to predict diabetic status without HbA1c, CpG24 methylation status and total nuclear methylation proved to be the most powerful predictors in both the binary (Fig. 5c) and multiple (Fig. 5d) classification datasets. Ultimately, both in the prediction of type 2 diabetes mellitus (Fig. 5e) and in assessing the onset (Fig. 5f), CpG24 hypomethylation was strongly correlated with total nuclear hypermethylation. CART tenfold cross validation confirmed findings for binary [testing (0.78), training (0.832)] and multiple [testing (0.67), training (0.542)] classification (Additional file 1: Figure S5A–D). Within our datasets, CpG24 methylation status and total nuclear methylation provided the best predictive measures for assessing type 2 diabetes mellitus. The incorporation of physiological, biochemical, genetic, and epigenetic features with machine-learning algorithms exemplifies the potential for more informative diagnostics in the future, as well as personalized approaches to generalized treatment modalities (Fig. 6).

## Discussion

Machine-learning can be applied as a systems biology approach, integrating multiple classes of biometric data to assess the importance of specific factors, while also predicting future outcomes. Whereas conventional assessments of disease identification exist, more detailed genomic and epigenomic testing is likely to reveal a comprehensive, systemic valuation of an etiology. To-date, studies have applied machine-learning algorithms in examining the physiological, biochemical, and/or genetic components of disease onset or progression [51]. The advantage of our current study is through the assimilation of patient-matched data across a variety of critically impacted systems, providing an archetype for developing novel, descriptive, diagnostic measures through machine-learning algorithms that are specific for each disease type. By individually representing our datasets in Figs. 2, 3 and 4, we were able to reach more conclusive data in Fig. 5 by choosing the most predictive features for our final model. For the first time, a multi-omics, machine-learning approach was used to assess the progression and development of type 2 diabetes mellitus in a patient population, identifying potential biomarkers for cardiovascular risk and revealing the fundamental role of genetics in the pathology.

### Molecular pathogenesis and machine-learning

While clinical practice has recently experienced a surge in deep learning applications used for non-invasive imaging [52], implementing machine-learning algorithms to the fundamental biochemistry and cellular and molecular processes of the body is now only blossoming. Onset and progression of type 2 diabetes has been traditionally measured through blood glucose levels, but, the multifaceted aspects of the disease could create variability in prognosis between vastly different demographic and ethnic groups. Owusu Adjah et al. [14] recently identified BMI as a risk factor for determining ethnic group disposition to type 2 diabetes mellitus. Specifically, the relationship between BMI and increased incidence of diabetes mellitus is non-linear; some groups, such as South Asian populations, were more disposed to developing the disease even at lower BMIs. While the current manuscript examines cardiovascular tissue, other less invasive approaches have been used to apply machine-learning algorithms. By retrieving blood from the basilica vein, circulating biomarkers were examined for their role in predicting early recurrence of atrial fibrillation following cryoballoon ablation [53]. Support vector machines confirmed that decreased levels of creatine-kinase (CK-MB) and Troponin T (TnT) were associated with increased early recurrence of atrial fibrillation following cryoballoon ablation. Additionally, a unique, non-invasive approach for potentially diagnosing type 2 diabetes in patients was performed through the examination of toenails. Carter et al. [54], through a variety of machine learning algorithms, focused on 22 elements, including aluminum, cesium, nickel, vanadium, and zinc, and was able to get an AUC of 0.90 when predicting diabetic status using a random forest model.

Similar to parts of the aims of this study, other groups have attempted to use machine learning to separate diabetic and non-diabetic patients without the inclusion of blood glucose or HbA1c [55]. In a testing set of 13,700 patients from the Luzhou, China region, random forest machine-learning algorithms provided a 0.7225 accuracy when predicting diabetic status from physical examination data in the absence of blood glucose [55]. Also using a random forest model, Tang et al. [56] revealed how CpG island methylation data, combined with microRNA expression profiles, can be instrumental in cancer pathogenesis; implementing this two-feature selection process, they were able to identify the best tissue specific features, ultimately allowing for the identification of the originating tissue where tumor progression began. In a similar fashion, the machine-learning algorithm HeteSim [57], which examines heterogeneous datasets and calculates their relatedness, was employed in ascribing how gene profiles can be related to phenotypic outcomes, specifically in the validation and prediction of genes classified within major diseases [58].

While understanding how to better form prognoses and treat cardiac dysfunction in patients with type 2 diabetes mellitus remains a critical mission, more than 80 million American adults, most of which are undiagnosed, are prediabetic [59]. In the current work, we have implemented predictive algorithms to assess biomarkers likely involved in the onset, as well as prediabetic progression, of type 2 diabetes mellitus. Although multiple classification categories further reduce the predictive power of the model, separation into distinct groupings revealed a unique phenotype for prediabetics (Fig. 4h). The effects of diabetes mellitus on the body is a high glucose stressed condition, altering substrate metabolism and causing systemic inflammation [60]. Due to this environmental change, researchers have shown how epigenetic changes occur across most, if not all, tissues that are impacted by diabetes mellitus [49, 61].

In the cardiovascular system, the heart, circulatory system, and regulating immune system are all transcriptionally regulated through epigenetic alterations [48, 62], resulting in cellular adaptations to the environmental stress. Examining atrial appendages, the results obtained in this study are a direct reflection of changes within the heart. While blood is more easily acquired in type 2 diabetic patients, cardiac tissue, which is mitochondrially rich, provides a direct connection between physiological dysfunction observed in the heart and the impact of altered genomic profiles in the mitochondrion and nucleus. Machine-learning, which at current has been applied to very few genetic applications, may play a significant role in defining the epigenome of those with diabetes mellitus, likely unveiling genes and molecular pathways first impacted by the pathology.

### The challenges of machine learning in the clinical setting

Machine-learning algorithms produce generalizations as they are inherently predictive, which means a smaller sample size can occasionally result in increased emphasis on outliers within the patient dataset and determination of the outliers’ biomarker features to be most influential in disease diagnosis. With a limited 50 patient dataset, there are concerns of overfitting the model, where the derived classification tree would have branches for each patient sample encountered during training. If this was to occur, the produced tree would fail future test cases while maintaining near perfect training accuracy, which was not observed. Tenfold cross validation ensured that no single developed tree was composed solely of outliers or a group of patient data of one label type, allowing patients of different labels to train the algorithm. Additionally, choosing seed values provided an even patient distribution during model training and testing. Both tenfold cross validation and setting a seed allowed the derived models to not over fit the training data. With this being said, it should be noted that the small sample size limits the conclusions and predictions made by the machine-learning algorithms within the manuscript, and future investigations will need to validate specific features, including CpG24 of TFAM and global nuclear DNA methylation.

For developed frameworks and the implemented SHAP visualization, the results are inherently regulated by HbA1c since patient HbA1c values were used to assign the labels from which the machine-learning algorithm then proceeded to train. HbA1c is used as a guide in this study to help clarify how clinically assessed progression of diabetes (commonly through HbA1c levels) is related to the biochemical and genetic signatures found in the heart. Although no specific biomarker or biomarker combinations can replace HbA1c due to the apparent diagnostic bias in this study (essentially ~ 100% accuracy when included), they can provide predictive accuracies near that of HbA1c. While previous clinical diagnoses determined a patients’ diabetic status in this study, some patients diagnosed as type 2 diabetics had HbA1c levels within normal ranges; begging the question of whether sustained, or attenuated, health effects can be accurately assessed through HbA1c levels alone when intervention (lifestyle or medicinally) occurs? Ultimately, this study provides a machine-learning algorithm utilizing the respective advantages of HbA1c in combination with other biomarkers to help circumvent the limitations of modern HbA1c diagnosis, as well as introduces completely novel cardiac risk stratification paradigms for patients with type 2 diabetes mellitus.

The quantity and diversity of omics-based approaches continues to expand. Convenience and increasingly inexpensive options for biometric-based valuations incite a growing demand for the incorporation and meaningful explanation of large and diverse patient datasets. The methodology outlined in this manuscript can serve as an archetype for the development and implementation of machine-learning to other disciplines seeking to evaluate disease progression. By using various health outcomes datasets, we were able to identify, and combine, the most prominent biomarkers into an accurate predictive algorithm engineered around 50 patients. While we have identified specific genetic features that are highly predictive in 50 patients, as a much larger patient population is applied to this model, the prioritization of other features is likely to occur, enhancing the diagnostic potential for the individual diabetic or prediabetic patient. Indeed, this is the advantage of using machine-learning models, in that they continue to learn and develop more accurate predictions as the number of features and sampled population grows.

## Conclusions

Our work highlights the importance of identifying biomarkers in systems known to be disturbed during the disease (i.e. the mitochondrion and nucleus), and further applying these biological systems to personalized prognostics. By implementing classification tree, machine-learning algorithms to cardiac tissue from type 2 diabetic patients, we determined that hypermethylation of the nuclear genome was predictive of diabetic status and that it may provide added benefit to diagnostic applications in the future. Additionally, through our machine-learning model, as little as a ~ 5% change in methylation status of a gene promoter could provide valuable predictive data when determining diabetic status. Defining new diagnostic parameters, better predicting future health outcomes, and specializing modalities of care begins with the integration of “big data” into machine-learning systems; this study reveals how integration of data assists in the determination of diabetic status in the heart.

## Additional file

**Additional file 1.** Supplemental data to the primary manuscript including, patient characteristics, primer design, and tenfold cross validation for machine learning algorithms. For each specific data set, we applied six different machine-learning models (CART, LR, LDA, KNN, NB, SVM) and determined which model would yield the best predictions on the data sets. CART yielded the best result, the other test/train accuracies are provided for comparison and support of our conclusions.

## Data Availability

The datasets generated and/or analysed during the current study, including sequencing files and computer code, are available (Refer to “Methods”, section “Data availability”). Primary used and/or analysed during the current study are available from the corresponding author on reasonable request.

## Abbreviations

5hmC: 5-hydroxymethylcytosine
5mC: 5-methylcytosine
AUC: area under the curve
CART: Classification and Regression Trees
CpG: cytosine nucleotide followed by a guanine nucleotide
ETC: electron transport chain
HbA1c: glycated hemoglobin
LR: Logistic Regression
LDA: Linear Discriminant Analysis
KNN: K-Nearest Neighbors
NB: Naive Bayes
SHAP: SHapley Additive exPlanations
SVM: Support Vector Machine
T2DM: type 2 diabetes mellitus
TFAM: transcription factor A, mitochondrial
