# Supplementary material for: Machine-learning to stratify diabetic patients using novel cardiac biomarkers and integrative genomics
Source: Cardiovasc Diabetol. 2019 Jun 11;18:78. doi: 10.1186/s12933-019-0879-0 (PMC6560734; doi:10.1186/s12933-019-0879-0)
Supplement: Supplementary file 1 — Additional file 1. Supplemental data to the primary manuscript including, patient characteristics, primer design, and tenfold cross validation for machine learning algorithms. For each specific data set, we applied six different machine-learning models (CART, LR, LDA, KNN, NB, SVM) and determined which model would yield the best predictions on the data sets. CART yielded the best result, the other test/train accuracies are provided for comparison and support of our conclusions. [file 12933_2019_879_MOESM1_ESM.docx]

**ADDITIONAL FILE**

**Machine Learning to Stratify Diabetic Patients using Novel Cardiac Biomarkers and Integrative Genomics**

Quincy A. Hathaway,^1,2^ Skyler M. Roth,^3^ Mark V. Pinti,^4^ Daniel C. Sprando,^5^ Amina Kunovac,^1,2^ Andrya J. Durr,^1,2^ Chris C. Cook,^6^ Garrett K. Fink,^1^ Tristen B. Cheuvront,^3^ Jasmine H. Grossman,^3^ Ghadah A. Aljahli,^3^ Andrew D. Taylor,^1,2^ Andrew P. Giromini,^5^ Jessica L. Allen,^3^ and *John M. Hollander^1,2^

**^1^**Division of Exercise Physiology, West Virginia University School of Medicine, Morgantown, WV, USA. **^2^**Mitochondria, Metabolism & Bioenergetics Working Group, West Virginia University School of Medicine, Morgantown, WV, USA. **^3^**Department of Chemical and Biomedical Engineering, West Virginia University, Morgantown, WV, USA. **^4^**West Virginia University School of Pharmacy, Morgantown, WV, USA. **^5^**West Virginia University School of Medicine, Morgantown, WV, USA. **^6^**Cardiovascular and Thoracic Surgery, West Virginia University School of Medicine, Morgantown, WV, USA.

*Corresponding Author:

John M. Hollander, Ph.D., F.A.H.A.

Division of Exercise Physiology

West Virginia University School of Medicine

PO Box 9227

1 Medical Center Drive

Morgantown, WV 26506

Tel: 1-(304) 293-3683

Fax: 1-(304) 293-7105

Email: [jhollander@hsc.wvu.edu](mailto:jhollander@hsc.wvu.edu)

**Additional Tables:**

**Table S1**

| **Parameter** | **Non-Diabetic** | **Pre-Diabetic** | **Type 2 Diabetic** |
| --- | --- | --- | --- |
| Age | 58.06 ± 3.692 | 66.43 ± 2.799 | 61.16 ± 3.047 |
| Sex | Male = 15, Female = 1 | Male = 11, Female = 3 | Male = 14, Female = 5 |
| BMI (kg/m^2^) | 30.11 ± 1.405 | 28.01 ± 1.669 | 29.14 ± 1.448 |
| Coronary Artery Disease | 68.75% ± 11.97% | 78.57% ± 11.38% | 100% ± 0.0% |
| Hypertension | 81.25% ± 10.08% | 92.86% ± 7.143% | 94.74% ± 5.263% |
| Valvular Disease | 31.25% ± 11.97% | 21.43% ± 11.38% | 15.79% ± 8.595% |
| HbA1c | 5.263 ± 0.08892 | 5.914 ± 0.0443 | **8.016 ± 0.4024*#** |

**Table S1:** Patient characteristics and demographic information for non-diabetic, pre-diabetic, and type 2 diabetic patients. Groups are considered significantly different if *P* ≤ 0.05 = ***** compared to non-diabetic or **#** compared to pre-diabetic. All data are presented as the mean ± standard error of the mean (SEM). HbA1c = glycated hemoglobin.

**Table S2**

| **Software** | **Purpose** | **Primer Name** | **Sequence** | **Product Size** |
| --- | --- | --- | --- | --- |
| MethPrimer 2.0 | Bisulfite Overhang PCR Sequencing | TFAM 1 F1 | TCGTCGGCAGCGTCAGATGTGTATAAGAGACAGGGGATAGAGGTGGTTTAATAG | 399 |
|  |  | TFAM 1 R1 | GTCTCGTGGGCTCGGAGATGTGTATAAGAGACAGATACACAACTCTACTCCAAACCTTC |  |
|  |  | TFAM 1 F2 | TCGTCGGCAGCGTCAGATGTGTATAAGAGACAGNGGGATAGAGGTGGTTTAATAG |  |
|  |  | TFAM 1 R2 | GTCTCGTGGGCTCGGAGATGTGTATAAGAGACAGNATACACAACTCTACTCCAAACCTTC |  |
|  |  | TFAM 1 F3 | TCGTCGGCAGCGTCAGATGTGTATAAGAGACAGNNGGGATAGAGGTGGTTTAATAG |  |
|  |  | TFAM 1 R3 | GTCTCGTGGGCTCGGAGATGTGTATAAGAGACAGNNATACACAACTCTACTCCAAACCTTC |  |
| MethPrimer 2.0 | Bisulfite Overhang PCR Sequencing | TFAM 2 F1 | TCGTCGGCAGCGTCAGATGTGTATAAGAGACAGAAAATGAAGATTAATGGGTTTTA | 356 |
|  |  | TFAM 2 R1 | GTCTCGTGGGCTCGGAGATGTGTATAAGAGACAGCTATTAAACCACCTCTATCCC |  |
|  |  | TFAM 2 F2 | TCGTCGGCAGCGTCAGATGTGTATAAGAGACAGNAAAATGAAGATTAATGGGTTTTA |  |
|  |  | TFAM 2 R2 | GTCTCGTGGGCTCGGAGATGTGTATAAGAGACAGNCTATTAAACCACCTCTATCCC |  |
|  |  | TFAM 2 F3 | TCGTCGGCAGCGTCAGATGTGTATAAGAGACAGNNAAAATGAAGATTAATGGGTTTTA |  |
|  |  | TFAM 2 R3 | GTCTCGTGGGCTCGGAGATGTGTATAAGAGACAGNNCTATTAAACCACCTCTATCCC |  |
| Primer3 | Chromatin Immunoprecipitation qPCR | D-Loop F1 | CATGGGGAAGCAGATTTGGG | 174 |
|  |  | D-Loop R1 | TTGTAAGCATGGGGAGGGG |  |
|  |  | D-Loop F2 | TACATTACTGCCAGCCACCA | 113 |
|  |  | D-Loop R2 | CATGGGGACGAGAAGGGATT |  |
|  |  | D-Loop F3 | CTTGACCACCATCCTCCGT | 137 |
|  |  | D-Loop R3 | AGAGCTCCCGTGAGTGGTTA |  |
|  |  | D-Loop F4 | GAGCTCTCCATGCATTTGGT | 91 |
|  |  | D-Loop R4 | TACTGCGACATAGGGTGCTC |  |
|  |  | D-Loop F5 | GAGCACCCTATGTCGCAGTA | 169 |
|  |  | D-Loop R5 | CTGTGTGGAAAGTGGCTGTG |  |
|  |  | D-Loop F6 | GTCTGCACAGCCACTTTCC | 225 |
|  |  | D-Loop R6 | TAGTATGGGAGTGGGAGGGG |  |
|  |  | D-Loop F7 | CCCCTCCCACTCCCATACTA | 183 |
|  |  | D-Loop R7 | GGTGATGTGAGCCCGTCTAA |  |

**Table S2:** Primer design. Using MethPrimer 2.0 for bisulfite converted DNA and Primer3 for chromatin immunoprecipitation, primers were designed to measure TFAM CpG island methylation and TFAM binding to the D-Loop region of the mitochondrial DNA, respectively. TFAM = transcription factor A, mitochondrial.

**Table S3**

| **SEED = 2** | | | | | |
| --- | --- | --- | --- | --- | --- |
|  | **CART Binary** | | | | |
|  | **Features Implemented in Training and Testing by Trial** | **Training (Average Accuracy)** | **Testing (F1 Score)** | **Train St.D Average** | **Test St. D** |
| TRIAL 1 | Nuc 5mC, Complex I, III, IV | **0.73** | **0.836** | **0.1778** | **0.092** |
| TRIAL 2 | Methyl, Complex III, IV, V | **0.6782** | **0.74** | **0.1902** | **0.055** |
| TRIAL 3 | Methyl, Nuc 5hm, Complex I | **0.67** | **0.67** | **0.2072** | **0.000** |
| TRIAL 4 | Complex I, III, IV | **0.573** | **0.482** | **0.2218** | **0.085** |
| TRIAL 5 | Methyl, Nuc 5mC, Complex I, Citrate Synthase | **0.7448** | **0.604** | **0.2012** | **0.054** |
| TRIAL 6 | Methyl, Complex I, III | **0.652** | **0.7** | **0.2252** | **0.000** |
|  |  |  |  |  |  |
|  | **LR Binary** | | | | |
|  | **Features Implemented in Training and Testing by Trial** | **Training (Average Accuracy)** | **Testing (F1 Score)** | **Train St.D Average** | **Test St. D** |
| TRIAL 1 | Nuc 5mC, Complex I, III, IV | **0.791667** | **0.7** | **0.107044** | **0** |
| TRIAL 2 | Methyl, Complex III, IV, V | **0.583333** | **0.4** | **0.182574** | **0** |
| TRIAL 3 | Methyl, Nuc 5hm, Complex I | **0.708333** | **0.52** | **0.201556** | **0** |
| TRIAL 4 | Complex I, III, IV | **0.608333** | **0.6** | **0.186525** | **0** |
| TRIAL 5 | Methyl, Nuc 5mC, Complex I, Citrate Synthase | **0.725** | **0.6** | **0.175** | **0** |
| TRIAL 6 | Methyl, Complex I, III | **0.633333** | **0.38** | **0.217945** | **0** |
|  |  |  |  |  |  |
|  | **LDA Binary** | | | | |
|  | **Features Implemented in Training and Testing by Trial** | **Training (Average Accuracy)** | **Testing (F1 Score)** | **Train St.D Average** | **Test St. D** |
| TRIAL 1 | Nuc 5mC, Complex I, III, IV | **0.741667** | **0.7** | **0.114564** | **0** |
| TRIAL 2 | Methyl, Complex III, IV, V | **0.558333** | **0.4** | **0.175** | **0** |
| TRIAL 3 | Methyl, Nuc 5hm, Complex I | **0.683333** | **0.7** | **0.210159** | **0** |
| TRIAL 4 | Complex I, III, IV | **0.608333** | **0.6** | **0.149304** | **0** |
| TRIAL 5 | Methyl, Nuc 5mC, Complex I, Citrate Synthase | **0.675** | **0.8** | **0.195256** | **0** |
| TRIAL 6 | Methyl, Complex I, III | **0.633333** | **0.49** | **0.187083** | **0** |
|  |  |  |  |  |  |
|  | **KNN Binary** | | | | |
|  | **Features Implemented in Training and Testing by Trial** | **Training (Average Accuracy)** | **Testing (F1 Score)** | **Train St.D Average** | **Test St. D** |
| TRIAL 1 | Nuc 5mC, Complex I, III, IV | **0.675** | **0.45** | **0.317214** | **0** |
| TRIAL 2 | Methyl, Complex III, IV, V | **0.825** | **0.2** | **0.195256** | **0** |
| TRIAL 3 | Methyl, Nuc 5hm, Complex I | **0.633333** | **0.67** | **0.15** | **0** |
| TRIAL 4 | Complex I, III, IV | **0.675** | **0.45** | **0.317214** | **0** |
| TRIAL 5 | Methyl, Nuc 5mC, Complex I, Citrate Synthase | **0.633333** | **0.67** | **0.15** | **0** |
| TRIAL 6 | Methyl, Complex I, III | **0.675** | **0.38** | **0.275** | **0** |
|  |  |  |  |  |  |
|  | **NB Binary** | | | | |
|  | **Features Implemented in Training and Testing by Trial** | **Training (Average Accuracy)** | **Testing (F1 Score)** | **Train St.D Average** | **Test St. D** |
| TRIAL 1 | Nuc 5mC, Complex I, III, IV | **0.7** | **0.38** | **0.217945** | **0** |
| TRIAL 2 | Methyl, Complex III, IV, V | **0.625** | **0.23** | **0.321131** | **0** |
| TRIAL 3 | Methyl, Nuc 5hm, Complex I | **0.65** | **0.7** | **0.229129** | **0** |
| TRIAL 4 | Complex I, III, IV | **0.6** | **0.29** | **0.229129** | **0** |
| TRIAL 5 | Methyl, Nuc 5mC, Complex I, Citrate Synthase | **0.675** | **0.7** | **0.275** | **0** |
| TRIAL 6 | Methyl, Complex I, III | **0.675** | **0.49** | **0.296859** | **0** |
|  |  |  |  |  |  |
|  | **SVM Binary** | | | | |
|  | **Features Implemented in Training and Testing by Trial** | **Training (Average Accuracy)** | **Testing (F1 Score)** | **Train St.D Average** | **Test St. D** |
| TRIAL 1 | Nuc 5mC, Complex I, III, IV | **0.633333** | **0.33** | **0.187083** | **0** |
| TRIAL 2 | Methyl, Complex III, IV, V | **0.633333** | **0.52** | **0.187083** | **0** |
| TRIAL 3 | Methyl, Nuc 5hm, Complex I | **0.608333** | **0.52** | **0.149304** | **0** |
| TRIAL 4 | Complex I, III, IV | **0.633333** | **0.33** | **0.187083** | **0** |
| TRIAL 5 | Methyl, Nuc 5mC, Complex I, Citrate Synthase | **0.658333** | **0.67** | **0.184278** | **0** |
| TRIAL 6 | Methyl, Complex I, III | **0.608333** | **0.52** | **0.149304** | **0** |

**Table S3:** Machine learning analyses for physiological/biochemical features using binary classification. CART = Classification and Regression Trees, LR = Logistic Regression, LDA = Linear Discriminant Analysis, KNN = K-Nearest Neighbors, NB = Naïve Bayes, SVM = Support Vector Machines. Nuc = Nuclear, Mito = Mitochondrial, 5mC = 5-methylcytosine, 5hmC = 5-hydroxymethylcytosine, Methyl = S-adenosyl methionine methyltransferase activity, St.D. = standard deviation, binary = non-diabetic and type 2 diabetic, multiple = non-diabetic, prediabetic, and type 2 diabetic.

**Table S4**

| **SEED = 22** | | | | | |
| --- | --- | --- | --- | --- | --- |
|  | **CART Multiple** | | | | |
|  | **Features Implemented in Training and Testing by Trial** | **Training (Average Accuracy)** | **Testing (F1 Score)** | **Train St.D Average** | **Test St. D** |
| TRIAL 1 | Mito 5hmC, Nuc 5mC, Complex I, III, V | **0.49** | **0.35** | **0.2968** | **0.000** |
| TRIAL 2 | Nuc 5mC, Complex I, III, IV, V, Citrate Synthase | **0.44** | **0.48** | **0.2828** | **0.079** |
| TRIAL 3 | Methyl, Complex III, V | **0.545** | **0.598** | **0.26** | **0.045** |
| TRIAL 4 | Nuc 5mC, Complex III, V | **0.52** | **0.412** | **0.2266** | **0.049** |
| TRIAL 5 | Methyl, Nuc 5mC, Citrate Synthase | **0.4532** | **0.464** | **0.2362** | **0.048** |
| TRIAL 6 | Methyl, Nuc 5mC, Complex III, Citrate Synthase | **0.4748** | **0.36** | **0.1812** | **0.060** |
|  |  |  |  |  |  |
|  | **LR Multiple** | | | | |
|  | **Features Implemented in Training and Testing by Trial** | **Training (Average Accuracy)** | **Testing (F1 Score)** | **Train St.D Average** | **Test St. D** |
| TRIAL 1 | Mito 5hmC ,Nuc 5mC, Complex I, III, V | **0.5** | **0.28** | **0.33541** | **0** |
| TRIAL 2 | Nuc 5mC, Complex I, III, IV, V, Citrate Synthase | **0.5** | **0.26** | **0.316228** | **0** |
| TRIAL 3 | Methyl, Complex III, V | **0.525** | **0.1** | **0.283945** | **0** |
| TRIAL 4 | Nuc 5mC, Complex III, V | **0.525** | **0.27** | **0.283945** | **0** |
| TRIAL 5 | Methyl, Nuc 5mC, Citrate Synthase | **0.45** | **0.22** | **0.291548** | **0** |
| TRIAL 6 | Methyl, Nuc 5mC, Complex III, Citrate Synthase | **0.5** | **0.12** | **0.295804** | **0** |
|  |  |  |  |  |  |
|  | **LDA Multiple** | | | | |
|  | **Features Implemented in Training and Testing by Trial** | **Training (Average Accuracy)** | **Testing (F1 Score)** | **Train St.D Average** | **Test St. D** |
| TRIAL 1 | Mito 5hmC ,Nuc 5mC, Complex I, III, V | **0.475** | **0.15** | **0.325** | **0** |
| TRIAL 2 | Nuc 5mC, Complex I, III, IV, V, Citrate Synthase | **0.525** | **0.32** | **0.283945** | **0** |
| TRIAL 3 | Methyl, Complex III, V | **0.6** | **0.11** | **0.339116** | **0** |
| TRIAL 4 | Nuc 5mC, Complex III, V | **0.55** | **0.28** | **0.331662** | **0** |
| TRIAL 5 | Methyl, Nuc 5mC, Citrate Synthase | **0.5** | **0.4** | **0.37081** | **0** |
| TRIAL 6 | Methyl, Nuc 5mC, Complex III, Citrate Synthase | **0.5** | **0.4** | **0.353553** | **0** |
|  |  |  |  |  |  |
|  | **KNN Multiple** | | | | |
|  | **Features Implemented in Training and Testing by Trial** | **Training (Average Accuracy)** | **Testing (F1 Score)** | **Train St.D Average** | **Test St. D** |
| TRIAL 1 | Mito 5hmC ,Nuc 5mC, Complex I, III, V | **0.55** | **0.1** | **0.291548** | **0** |
| TRIAL 2 | Nuc 5mC, Complex I, III, IV, V, Citrate Synthase | **0.575** | **0.15** | **0.296859** | **0** |
| TRIAL 3 | Methyl, Complex III, V | **0.516667** | **0.1** | **0.229129** | **0** |
| TRIAL 4 | Nuc 5mC, Complex III, V | **0.516667** | **0.1** | **0.229129** | **0** |
| TRIAL 5 | Methyl, Nuc 5mC, Citrate Synthase | **0.425** | **0.23** | **0.336341** | **0** |
| TRIAL 6 | Methyl, Nuc 5mC, Complex III, Citrate Synthase | **0.491667** | **0.3** | **0.215542** | **0** |
|  |  |  |  |  |  |
|  | **NB Multiple** | | | | |
|  | **Features Implemented in Training and Testing by Trial** | **Training (Average Accuracy)** | **Testing (F1 Score)** | **Train St.D Average** | **Test St. D** |
| TRIAL 1 | Mito 5hmC ,Nuc 5mC, Complex I, III, V | **0.4** | **0.15** | **0.3** | **0** |
| TRIAL 2 | Nuc 5mC, Complex I, III, IV, V, Citrate Synthase | **0.45** | **0.22** | **0.269258** | **0** |
| TRIAL 3 | Methyl, Complex III, V | **0.6** | **0.1** | **0.254951** | **0** |
| TRIAL 4 | Nuc 5mC, Complex III, V | **0.475** | **0.15** | **0.283945** | **0** |
| TRIAL 5 | Methyl, Nuc 5mC, Citrate Synthase | **0.491667** | **0.48** | **0.267317** | **0** |
| TRIAL 6 | Methyl, Nuc 5mC, Complex III, Citrate Synthase | **0.475** | **0.22** | **0.378319** | **0** |
|  |  |  |  |  |  |
|  | **SVM Multiple** | | | | |
|  | **Features Implemented in Training and Testing by Trial** | **Training (Average Accuracy)** | **Testing (F1 Score)** | **Train St.D Average** | **Test St. D** |
| TRIAL 1 | Mito 5hmC ,Nuc 5mC, Complex I, III, V | **0.366667** | **0.14** | **0.217945** | **0** |
| TRIAL 2 | Nuc 5mC, Complex I, III, IV, V, Citrate Synthase | **0.416667** | **0.14** | **0.241523** | **0** |
| TRIAL 3 | Methyl, Complex III, V | **0.366667** | **0.14** | **0.269258** | **0** |
| TRIAL 4 | Nuc 5mC, Complex III, V | **0.366667** | **0.14** | **0.269258** | **0** |
| TRIAL 5 | Methyl, Nuc 5mC, Citrate Synthase | **0.5** | **0.23** | **0.316228** | **0** |
| TRIAL 6 | Methyl, Nuc 5mC, Complex III, Citrate Synthase | **0.466667** | **0.48** | **0.298608** | **0** |

**Table S4:** Machine learning analyses for physiological/biochemical features using multiple classification. CART = Classification and Regression Trees, LR = Logistic Regression, LDA = Linear Discriminant Analysis, KNN = K-Nearest Neighbors, NB = Naïve Bayes, SVM = Support Vector Machines. Nuc = Nuclear, Mito = Mitochondrial, 5mC = 5-methylcytosine, 5hmC = 5-hydroxymethylcytosine, Methyl = S-adenosyl methionine methyltransferase activity, St.D. = standard deviation, binary = non-diabetic and type 2 diabetic, multiple = non-diabetic, prediabetic, and type 2 diabetic.

**Table S5**

| SEED = 16 | | | | | |
| --- | --- | --- | --- | --- | --- |
|  | **CART Binary** | | | | |
|  | **Features Implemented in Training and Testing by Trial** | **Training (Average Accuracy)** | **Testing (F1 Score)** | **Train St.D Average** | **Test St. D** |
| TRIAL 1 | 72, 310, 3010, 4769, 8697, 11419, 11812, 14233, 15904, 16362 | **0.92** | **0.70** | **0.17** | **0** |
| TRIAL 2 | 310, 3010, 4769, 11419, 16362 | **0.92** | **0.58** | **0.17** | **0** |
| TRIAL 3 | 310, 4769, 16362 | **0.77** | **0.58** | **0.18** | **0** |
| TRIAL 4 | 72, 310, 16362 | **0.72** | **0.79** | **0.31** | **0** |
|  |  |  |  |  |  |
|  | **LR Binary** | | | | |
|  | **Features Implemented in Training and Testing by Trial** | **Training (Average Accuracy)** | **Testing (F1 Score)** | **Train St.D Average** | **Test St. D** |
| TRIAL 1 | 72, 310, 3010, 4769, 8697, 11419, 11812, 14233, 15904, 16362 | **0.7833** | **0.67** | **0.2506** | **0** |
| TRIAL 2 | 310, 3010, 4769, 11419, 16362 | **0.7833** | **0.67** | **0.2506** | **0** |
| TRIAL 3 | 310, 4769, 16362 | **0.7833** | **0.67** | **0.2506** | **0** |
| TRIAL 4 | 72, 310, 16362 | **0.7833** | **0.67** | **0.2506** | **0** |
|  |  |  |  |  |  |
|  | **LDA Binary** | | | | |
|  | **Features Implemented in Training and Testing by Trial** | **Training (Average Accuracy)** | **Testing (F1 Score)** | **Train St.D Average** | **Test St. D** |
| TRIAL 1 | 72, 310, 3010, 4769, 8697, 11419, 11812, 14233, 15904, 16362 | **0.8083** | **0.58** | **0.2983** | **0** |
| TRIAL 2 | 310, 3010, 4769, 11419, 16362 | **0.8917** | **0.58** | **0.175** | **0** |
| TRIAL 3 | 310, 4769, 16362 | **0.7083** | **0.67** | **0.2083** | **0** |
| TRIAL 4 | 72, 310, 16362 | **0.75** | **0.67** | **0.2838** | **0** |
|  |  |  |  |  |  |
|  | **KNN Binary** | | | | |
|  | **Features Implemented in Training and Testing by Trial** | **Training (Average Accuracy)** | **Testing (F1 Score)** | **Train St.D Average** | **Test St. D** |
| TRIAL 1 | 72, 310, 3010, 4769, 8697, 11419, 11812, 14233, 15904, 16362 | **0.8917** | **0.45** | **0.175** | **0** |
| TRIAL 2 | 310, 3010, 4769, 11419, 16362 | **0.8917** | **0.45** | **0.175** | **0** |
| TRIAL 3 | 310, 4769, 16362 | **0.7583** | **0.52** | **0.2399** | **0** |
| TRIAL 4 | 72, 310, 16362 | **0.7333** | **0.33** | **0.2261** | **0** |
|  |  |  |  |  |  |
|  | **NB Binary** | | | | |
|  | **Features Implemented in Training and Testing by Trial** | **Training (Average Accuracy)** | **Testing (F1 Score)** | **Train St.D Average** | **Test St. D** |
| TRIAL 1 | 72, 310, 3010, 4769, 8697, 11419, 11812, 14233, 15904, 16362 | **0.775** | **0.67** | **0.2473** | **0** |
| TRIAL 2 | 310, 3010, 4769, 11419, 16362 | **0.7833** | **0.67** | **0.2506** | **0** |
| TRIAL 3 | 310, 4769, 16362 | **0.7833** | **0.67** | **0.2506** | **0** |
| TRIAL 4 | 72, 310, 16362 | **0.7833** | **0.67** | **0.2506** | **0** |
|  |  |  |  |  |  |
|  | **SVM Binary** | | | | |
|  | **Features Implemented in Training and Testing by Trial** | **Training (Average Accuracy)** | **Testing (F1 Score)** | **Train St.D Average** | **Test St. D** |
| TRIAL 1 | 72, 310, 3010, 4769, 8697, 11419, 11812, 14233, 15904, 16362 | **0.6** | **0.33** | **0.2409** | **0** |
| TRIAL 2 | 310, 3010, 4769, 11419, 16362 | **0.7833** | **0.67** | **0.2506** | **0** |
| TRIAL 3 | 310, 4769, 16362 | **0.7833** | **0.67** | **0.2506** | **0** |
| TRIAL 4 | 72, 310, 16362 | **0.7833** | **0.67** | **0.2506** | **0** |

**Table S5:** Machine learning analyses for mitochondrial SNP features using binary classification. CART = Classification and Regression Trees, LR = Logistic Regression, LDA = Linear Discriminant Analysis, KNN = K-Nearest Neighbors, NB = Naïve Bayes, SVM = Support Vector Machines. St.D. = standard deviation, binary = non-diabetic and type 2 diabetic, multiple = non-diabetic, prediabetic, and type 2 diabetic.

**Table S6**

| SEED = 87 | | | | | |
| --- | --- | --- | --- | --- | --- |
|  | **CART Multiple** | | | | |
|  | **Features Implemented in Training and Testing by Trial** | **Training (Average Accuracy)** | **Testing (F1 Score)** | **Train St.D Average** | **Test St. D** |
| TRIAL 1 | 114, 143, 151, 195, 310, 3010, 4295, 4769, 7028, 8860, 9055, 9840, 10398, 11812, 12246, 13401, 14766, 15191, 15223, 16124, 16183, 16189, 16270, 16336, 16344, 16362, 16390, 16519 | **0.67** | **0.576** | **0.23** | **0.009** |
| TRIAL 2 | 4295, 7028, 10398, 16362, 16390 | **0.808** | **0.5** | **0.171** | **0** |
| TRIAL 3 | 7028, 10398, 16362 | **0.758** | **0.5** | **0.181** | **0** |
| TRIAL 4 | 114, 143, 151, 195, 310, 16124, 16183, 16189, 16270, 16336, 16344, 16362, 16390, 16519 | **0.33** | **0.12** | **0.309** | **0** |
|  |  |  |  |  |  |
|  | **LR Multiple** | | | | |
|  | **Features Implemented in Training and Testing by Trial** | **Training (Average Accuracy)** | **Testing (F1 Score)** | **Train St.D Average** | **Test St. D** |
| TRIAL 1 | 114, 143, 151, 195, 310, 3010, 4295, 4769, 7028, 8860, 9055, 9840, 10398, 11812, 12246, 13401, 14766, 15191, 15223, 16124, 16183, 16189, 16270, 16336, 16344, 16362, 16390, 16519 | **0.6667** | **0.38** | **0.2789** | **0** |
| TRIAL 2 | 4295, 7028, 10398, 16362, 16390 | **0.6167** | **0.5** | **0.2333** | **0** |
| TRIAL 3 | 7028, 10398, 16362 | **0.6417** | **0.5** | **0.2329** | **0** |
| TRIAL 4 | 114, 143, 151, 195, 310, 16124, 16183, 16189, 16270, 16336, 16344, 16362, 16390, 16519 | **0.3917** | **0.15** | **0.2238** | **0** |
|  |  |  |  |  |  |
|  | **LDA Multiple** | | | | |
|  | **Features Implemented in Training and Testing by Trial** | **Training (Average Accuracy)** | **Testing (F1 Score)** | **Train St.D Average** | **Test St. D** |
| TRIAL 1 | 114, 143, 151, 195, 310, 3010, 4295, 4769, 7028, 8860, 9055, 9840, 10398, 11812, 12246, 13401, 14766, 15191, 15223, 16124, 16183, 16189, 16270, 16336, 16344, 16362, 16390, 16519 | **0.575** | **0.58** | **0.2621** | **0** |
| TRIAL 2 | 4295, 7028, 10398, 16362, 16390 | **0.7833** | **0.5** | **0.159** | **0** |
| TRIAL 3 | 7028, 10398, 16362 | **0.7583** | **0.5** | **0.1805** | **0** |
| TRIAL 4 | 114, 143, 151, 195, 310, 16124, 16183, 16189, 16270, 16336, 16344, 16362, 16390, 16519 | **0.3833** | **0.15** | **0.2533** | **0** |
|  |  |  |  |  |  |
|  | **KNN Multiple** | | | | |
|  | **Features Implemented in Training and Testing by Trial** | **Training (Average Accuracy)** | **Testing (F1 Score)** | **Train St.D Average** | **Test St. D** |
| TRIAL 1 | 114, 143, 151, 195, 310, 3010, 4295, 4769, 7028, 8860, 9055, 9840, 10398, 11812, 12246, 13401, 14766, 15191, 15223, 16124, 16183, 16189, 16270, 16336, 16344, 16362, 16390, 16519 | **0.5417** | **0.25** | **0.2451** | **0** |
| TRIAL 2 | 4295, 7028, 10398, 16362, 16390 | **0.7583** | **0.5** | **0.1805** | **0** |
| TRIAL 3 | 7028, 10398, 16362 | **0.7583** | **0.5** | **0.1805** | **0** |
| TRIAL 4 | 114, 143, 151, 195, 310, 16124, 16183, 16189, 16270, 16336, 16344, 16362, 16390, 16519 | **0.35** | **0.14** | **0.2784** | **0** |
|  |  |  |  |  |  |
|  | **NB Multiple** | | | | |
|  | **Features Implemented in Training and Testing by Trial** | **Training (Average Accuracy)** | **Testing (F1 Score)** | **Train St.D Average** | **Test St. D** |
| TRIAL 1 | 114, 143, 151, 195, 310, 3010, 4295, 4769, 7028, 8860, 9055, 9840, 10398, 11812, 12246, 13401, 14766, 15191, 15223, 16124, 16183, 16189, 16270, 16336, 16344, 16362, 16390, 16519 | **0.45** | **0.35** | **0.2303** | **0** |
| TRIAL 2 | 4295, 7028, 10398, 16362, 16390 | **0.6167** | **0.68** | **0.2048** | **0** |
| TRIAL 3 | 7028, 10398, 16362 | **0.5417** | **0.68** | **0.1873** | **0** |
| TRIAL 4 | 114, 143, 151, 195, 310, 16124, 16183, 16189, 16270, 16336, 16344, 16362, 16390, 16519 | **0.3917** | **0.46** | **0.3119** | **0** |
|  |  |  |  |  |  |
|  | **SVM Multiple** | | | | |
|  | **Features Implemented in Training and Testing by Trial** | **Training (Average Accuracy)** | **Testing (F1 Score)** | **Train St.D Average** | **Test St. D** |
| TRIAL 1 | 114, 143, 151, 195, 310, 3010, 4295, 4769, 7028, 8860, 9055, 9840, 10398, 11812, 12246, 13401, 14766, 15191, 15223, 16124, 16183, 16189, 16270, 16336, 16344, 16362, 16390, 16519 | **0.45** | **0.14** | **0.2014** | **0** |
| TRIAL 2 | 4295, 7028, 10398, 16362, 16390 | **0.4917** | **0.14** | **0.1557** | **0** |
| TRIAL 3 | 7028, 10398, 16362 | **0.5417** | **0.3** | **0.1502** | **0** |
| TRIAL 4 | 114, 143, 151, 195, 310, 16124, 16183, 16189, 16270, 16336, 16344, 16362, 16390, 16519 | **0.45** | **0.14** | **0.2014** | **0** |

**Table S6:** Machine learning analyses for mitochondrial SNP features using multiple classification. CART = Classification and Regression Trees, LR = Logistic Regression, LDA = Linear Discriminant Analysis, KNN = K-Nearest Neighbors, NB = Naïve Bayes, SVM = Support Vector Machines. St.D. = standard deviation, binary = non-diabetic and type 2 diabetic, multiple = non-diabetic, prediabetic, and type 2 diabetic.

**Table S7**

| SEED = 8 | | | | | |
| --- | --- | --- | --- | --- | --- |
|  | **CART Binary** | | | | |
|  | **Features Implemented in Training and Testing by Trial** | **Training (Average Accuracy)** | **Testing (F1 Score)** | **Train St.D Average** | **Test St. D** |
| TRIAL 1 | CpG 24, CpG 26, CpG 28, CpG 29, CpG 39 | **0.86336** | **0.742** | **0.17244** | **0.06572671** |
| TRIAL 2 | CpG 24, CpG 28, CpG 29, CpG 39 | **0.88336** | **0.718** | **0.15724** | **0.06572671** |
| TRIAL 3 | CpG 24, CpG 26, CpG 28, CpG 29 | **0.89002** | **0.79** | **0.12708** | **0** |
| TRIAL 4 | CpG 24, CpG 28, CpG 29 | **0.925** | **0.79** | **0.1146** | **0** |
| TRIAL 5 | CpG 24, CpG 29 | **0.9** | **0.67** | **0.1225** | **0** |
|  |  |  |  |  |  |
|  | **LR Binary** | | | | |
|  | **Features Implemented in Training and Testing by Trial** | **Training (Average Accuracy)** | **Testing (F1 Score)** | **Train St.D Average** | **Test St. D** |
| TRIAL 1 | CpG 24, CpG 26, CpG 28, CpG 29, CpG 39 | **0.733333** | **0.49** | **0.189297** | **0** |
| TRIAL 2 | CpG 24, CpG 28, CpG 29, CpG 39 | **0.791667** | **0.6** | **0.190941** | **0** |
| TRIAL 3 | CpG 24, CpG 26, CpG 28, CpG 29 | **0.775** | **0.7** | **0.190212** | **0** |
| TRIAL 4 | CpG 24, CpG 28, CpG 29 | **0.833333** | **0.58** | **0.139443** | **0** |
| TRIAL 5 | CpG 24, CpG 29 | **0.766667** | **0.8** | **0.238048** | **0** |
|  |  |  |  |  |  |
|  | **LDA Binary** | | | | |
|  | **Features Implemented in Training and Testing by Trial** | **Training (Average Accuracy)** | **Testing (F1 Score)** | **Train St.D Average** | **Test St. D** |
| TRIAL 1 | CpG 24, CpG 26, CpG 28, CpG 29, CpG 39 | **0.758333** | **0.58** | **0.141667** | **0** |
| TRIAL 2 | CpG 24, CpG 28, CpG 29, CpG 39 | **0.808333** | **0.58** | **0.129368** | **0** |
| TRIAL 3 | CpG 24, CpG 26, CpG 28, CpG 29 | **0.758333** | **0.58** | **0.087003** | **0** |
| TRIAL 4 | CpG 24, CpG 28, CpG 29 | **0.808333** | **0.58** | **0.129368** | **0** |
| TRIAL 5 | CpG 24, CpG 29 | **0.733333** | **0.58** | **0.226078** | **0** |
|  |  |  |  |  |  |
|  | **KNN Binary** | | | | |
|  | **Features Implemented in Training and Testing by Trial** | **Training (Average Accuracy)** | **Testing (F1 Score)** | **Train St.D Average** | **Test St. D** |
| TRIAL 1 | CpG 24, CpG 26, CpG 28, CpG 29, CpG 39 | **0.625** | **0.45** | **0.214897** | **0** |
| TRIAL 2 | CpG 24, CpG 28, CpG 29, CpG 39 | **0.65** | **0.49** | **0.181812** | **0** |
| TRIAL 3 | CpG 24, CpG 26, CpG 28, CpG 29 | **0.683333** | **0.45** | **0.177951** | **0** |
| TRIAL 4 | CpG 24, CpG 28, CpG 29 | **0.783333** | **0.7** | **0.113039** | **0** |
| TRIAL 5 | CpG 24, CpG 29 | **0.858333** | **0.9** | **0.182764** | **0** |
|  |  |  |  |  |  |
|  | **NB Binary** | | | | |
|  | **Features Implemented in Training and Testing by Trial** | **Training (Average Accuracy)** | **Testing (F1 Score)** | **Train St.D Average** | **Test St. D** |
| TRIAL 1 | CpG 24, CpG 26, CpG 28, CpG 29, CpG 39 | **0.7** | **0.52** | **0.201384** | **0** |
| TRIAL 2 | CpG 24, CpG 28, CpG 29, CpG 39 | **0.7** | **0.52** | **0.201384** | **0** |
| TRIAL 3 | CpG 24, CpG 26, CpG 28, CpG 29 | **0.758333** | **0.52** | **0.234076** | **0** |
| TRIAL 4 | CpG 24, CpG 28, CpG 29 | **0.783333** | **0.52** | **0.194365** | **0** |
| TRIAL 5 | CpG 24, CpG 29 | **0.783333** | **0.52** | **0.224227** | **0** |
|  |  |  |  |  |  |
|  | **SVM Binary** | | | | |
|  | **Features Implemented in Training and Testing by Trial** | **Training (Average Accuracy)** | **Testing (F1 Score)** | **Train St.D Average** | **Test St. D** |
| TRIAL 1 | CpG 24, CpG 26, CpG 28, CpG 29, CpG 39 | **0.625** | **0.7** | **0.289276** | **0** |
| TRIAL 2 | CpG 24, CpG 28, CpG 29, CpG 39 | **0.716667** | **0.6** | **0.236291** | **0** |
| TRIAL 3 | CpG 24, CpG 26, CpG 28, CpG 29 | **0.783333** | **0.58** | **0.217945** | **0** |
| TRIAL 4 | CpG 24, CpG 28, CpG 29 | **0.783333** | **0.58** | **0.15899** | **0** |
| TRIAL 5 | CpG 24, CpG 29 | **0.783333** | **0.67** | **0.194365** | **0** |

**Table S7:** Machine learning analyses for Transcription Factor A, Mitochondrial (TFAM) CpG island methylation features using binary classification. CART = Classification and Regression Trees, LR = Logistic Regression, LDA = Linear Discriminant Analysis, KNN = K-Nearest Neighbors, NB = Naïve Bayes, SVM = Support Vector Machines. CpG = cytosine nucleotide followed by a guanine nucleotide, St.D. = standard deviation, binary = non-diabetic and type 2 diabetic, multiple = non-diabetic, prediabetic, and type 2 diabetic.

**Table S8**

| SEED = 22 | | | | | |
| --- | --- | --- | --- | --- | --- |
|  | **CART Multiple** | | | | |
|  | **Features Implemented in Training and Testing by Trial** | **Training (Average Accuracy)** | **Testing (F1 Score)** | **Train St.D Average** | **Test St. D** |
| TRIAL 1 | CpG 1, CpG 5, CpG 14, CpG 20, CpG 23, CpG 24, CpG 33, TFAM CpG | **0.71836** | **0.668** | **0.19942** | **0.05761944** |
| TRIAL 2 | CpG 1, CpG 14, CpG 20, CpG 23, CpG 24, CpG 33, TFAM CpG | **0.69832** | **0.666** | **0.20908** | **0.06024948** |
| TRIAL 3 | CpG 1, CpG 20, CpG 23, CpG 24, TFAM CpG | **0.73168** | **0.6** | **0.19586** | **0** |
| TRIAL 4 | CpG 1, CpG 14, CpG 20, CpG 23, CpG 24, CpG 35, TFAM CpG | **0.76668** | **0.644** | **0.21556** | **0.06024948** |
| TRIAL 5 | CpG 1, CpG 20, CpG 23, CpG 24, CpG 35, TFAM CpG | **0.74834** | **0.644** | **0.19818** | **0.06024948** |
|  |  |  |  |  |  |
|  | **LR Multiple** | | | | |
|  | **Features Implemented in Training and Testing by Trial** | **Training (Average Accuracy)** | **Testing (F1 Score)** | **Train St.D Average** | **Test St. D** |
| TRIAL 1 | CpG 1, CpG 5, CpG 14, CpG 20, CpG 23, CpG 24, CpG 33, TFAM CpG | **0.491667** | **0.15** | **0.242813** | **0** |
| TRIAL 2 | CpG 1, CpG 14, CpG 20, CpG 23, CpG 24, CpG 33, TFAM CpG | **0.491667** | **0.26** | **0.215542** | **0** |
| TRIAL 3 | CpG 1, CpG 20, CpG 23, CpG 24, TFAM CpG | **0.475** | **0.28** | **0.190212** | **0** |
| TRIAL 4 | CpG 1, CpG 14, CpG 20, CpG 23, CpG 24, CpG 35, TFAM CpG | **0.491667** | **0.26** | **0.242813** | **0** |
| TRIAL 5 | CpG 1, CpG 20, CpG 23, CpG 24, CpG 35, TFAM CpG | **0.466667** | **0.27** | **0.253311** | **0** |
|  |  |  |  |  |  |
|  | **LDA Multiple** | | | | |
|  | **Features Implemented in Training and Testing by Trial** | **Training (Average Accuracy)** | **Testing (F1 Score)** | **Train St.D Average** | **Test St. D** |
| TRIAL 1 | CpG 1, CpG 5, CpG 14, CpG 20, CpG 23, CpG 24, CpG 33, TFAM CpG | **0.466667** | **0.22** | **0.227303** | **0** |
| TRIAL 2 | CpG 1, CpG 14, CpG 20, CpG 23, CpG 24, CpG 33, TFAM CpG | **0.466667** | **0.28** | **0.227303** | **0** |
| TRIAL 3 | CpG 1, CpG 20, CpG 23, CpG 24, TFAM CpG | **0.475** | **0.28** | **0.190212** | **0** |
| TRIAL 4 | CpG 1, CpG 14, CpG 20, CpG 23, CpG 24, CpG 35, TFAM CpG | **0.441667** | **0.28** | **0.283945** | **0** |
| TRIAL 5 | CpG 1, CpG 20, CpG 23, CpG 24, CpG 35, TFAM CpG | **0.441667** | **0.28** | **0.283945** | **0** |
|  |  |  |  |  |  |
|  | **KNN Multiple** | | | | |
|  | **Features Implemented in Training and Testing by Trial** | **Training (Average Accuracy)** | **Testing (F1 Score)** | **Train St.D Average** | **Test St. D** |
| TRIAL 1 | CpG 1, CpG 5, CpG 14, CpG 20, CpG 23, CpG 24, CpG 33, TFAM CpG | **0.5** | **0.24** | **0.235702** | **0** |
| TRIAL 2 | CpG 1, CpG 14, CpG 20, CpG 23, CpG 24, CpG 33, TFAM CpG | **0.475** | **0.31** | **0.247347** | **0** |
| TRIAL 3 | CpG 1, CpG 20, CpG 23, CpG 24, TFAM CpG | **0.45** | **0.38** | **0.167498** | **0** |
| TRIAL 4 | CpG 1, CpG 14, CpG 20, CpG 23, CpG 24, CpG 35, TFAM CpG | **0.308333** | **0.38** | **0.186525** | **0** |
| TRIAL 5 | CpG 1, CpG 20, CpG 23, CpG 24, CpG 35, TFAM CpG | **0.416667** | **0.38** | **0.247207** | **0** |
|  |  |  |  |  |  |
|  | **NB Multiple** | | | | |
|  | **Features Implemented in Training and Testing by Trial** | **Training (Average Accuracy)** | **Testing (F1 Score)** | **Train St.D Average** | **Test St. D** |
| TRIAL 1 | CpG 1, CpG 5, CpG 14, CpG 20, CpG 23, CpG 24, CpG 33, TFAM CpG | **0.45** | **0.13** | **0.124722** | **0** |
| TRIAL 2 | CpG 1, CpG 14, CpG 20, CpG 23, CpG 24, CpG 33, TFAM CpG | **0.475** | **0.26** | **0.153885** | **0** |
| TRIAL 3 | CpG 1, CpG 20, CpG 23, CpG 24, TFAM CpG | **0.55** | **0.11** | **0.256038** | **0** |
| TRIAL 4 | CpG 1, CpG 14, CpG 20, CpG 23, CpG 24, CpG 35, TFAM CpG | **0.45** | **0.28** | **0.201384** | **0** |
| TRIAL 5 | CpG 1, CpG 20, CpG 23, CpG 24, CpG 35, TFAM CpG | **0.5** | **0.28** | **0.207498** | **0** |
|  |  |  |  |  |  |
|  | **SVM Multiple** | | | | |
|  | **Features Implemented in Training and Testing by Trial** | **Training (Average Accuracy)** | **Testing (F1 Score)** | **Train St.D Average** | **Test St. D** |
| TRIAL 1 | CpG 1, CpG 5, CpG 14, CpG 20, CpG 23, CpG 24, CpG 33, TFAM CpG | **0.5** | **0.07** | **0.260875** | **0** |
| TRIAL 2 | CpG 1, CpG 14, CpG 20, CpG 23, CpG 24, CpG 33, TFAM CpG | **0.483333** | **0.16** | **0.235112** | **0** |
| TRIAL 3 | CpG 1, CpG 20, CpG 23, CpG 24, TFAM CpG | **0.425** | **0.17** | **0.237024** | **0** |
| TRIAL 4 | CpG 1, CpG 14, CpG 20, CpG 23, CpG 24, CpG 35, TFAM CpG | **0.508333** | **0.07** | **0.248468** | **0** |
| TRIAL 5 | CpG 1, CpG 20, CpG 23, CpG 24, CpG 35, TFAM CpG | **0.508333** | **0.17** | **0.272463** | **0** |

**Table S8:** Machine learning analyses for Transcription Factor A, Mitochondrial (TFAM) CpG island methylation features using multiple classification. CART = Classification and Regression Trees, LR = Logistic Regression, LDA = Linear Discriminant Analysis, KNN = K-Nearest Neighbors, NB = Naïve Bayes, SVM = Support Vector Machines. CpG = cytosine nucleotide followed by a guanine nucleotide, TFAM CpG = total CpG methylation of the TFAM promoter region, St.D. = standard deviation, binary = non-diabetic and type 2 diabetic, multiple = non-diabetic, prediabetic, and type 2 diabetic.

**Table S9**

| SEED = 15 | | | | | |
| --- | --- | --- | --- | --- | --- |
|  | **CART Binary** | | | | |
|  | **Features Implemented in Training and Testing by Trial** | **Training (Average Accuracy)** | **Testing (F1 Score)** | **Train St.D Average** | **Test St. D** |
| TRIAL 1 | Methyl, Mito 5hmC, Nuc 5hmC, Mito 5mC, Nuc 5mC, CpG 11, CpG 23, CpG 24, CpG 28 | **0.786666** | **0.758** | **0.2043988** | **0.0491935** |
| TRIAL 2 | Methyl, Mito 5hmC, Nuc 5hmC, Mito 5mC, Nuc 5mC, CpG 1 - 24, TFAM CpG, TFAM Non-CpG | **0.8083318** | **0.714** | **0.2168344** | **0.06024948** |
| TRIAL 3 | Methyl, Mito 5hmC, Nuc 5hmC, Mito 5mC, Nuc 5mC, CpG 24, TFAM CpG, TFAM Non-CpG | **0.831667** | **0.67** | **0.2166188** | **0** |
| TRIAL 4 | Methyl, Nuc 5mC, CpG 1 - 24 | **0.7899998** | **0.736** | **0.209388** | **0.06024948** |
| TRIAL 5 | Nuc 5mC, CpG 1,CpG 7,CpG 8,CpG 9,CpG 11,CpG 12,CpG 22,CpG 23,CpG 24,CpG 28 | **0.743333** | **0.78** | **0.1964948** | **0** |
|  |  |  |  |  |  |
|  | **LR Binary** | | | | |
|  | **Features Implemented in Training and Testing by Trial** | **Training (Average Accuracy)** | **Testing (F1 Score)** | **Train St.D Average** | **Test St. D** |
| TRIAL 1 | Methyl, Mito 5hmC, Nuc 5hmC, Mito 5mC, Nuc 5mC, CpG 11, CpG 23, CpG 24, CpG 28 | **0.7** | **0.56** | **0.279384** | **0** |
| TRIAL 2 | Methyl, Mito 5hmC, Nuc 5hmC, Mito 5mC, Nuc 5mC, CpG 1 - 24, TFAM CpG, TFAM Non-CpG | **0.633333** | **0.67** | **0.208167** | **0** |
| TRIAL 3 | Methyl, Mito 5hmC, Nuc 5hmC, Mito 5mC, Nuc 5mC, CpG 24, TFAM CpG, TFAM Non-CpG | **0.758333** | **0.66** | **0.18047** | **0** |
| TRIAL 4 | Methyl, Nuc 5mC, CpG 1 - 24 | **0.683333** | **0.66** | **0.2** | **0** |
| TRIAL 5 | Nuc 5mC, CpG 1,CpG 7,CpG 8,CpG 9,CpG 11,CpG 12,CpG 22,CpG 23,CpG 24,CpG 28 | **0.658333** | **0.66** | **0.253996** | **0** |
|  |  |  |  |  |  |
|  | **LDA Binary** | | | | |
|  | **Features Implemented in Training and Testing by Trial** | **Training (Average Accuracy)** | **Testing (F1 Score)** | **Train St.D Average** | **Test St. D** |
| TRIAL 1 | Methyl, Mito 5hmC, Nuc 5hmC, Mito 5mC, Nuc 5mC, CpG 11, CpG 23, CpG 24, CpG 28 | **0.766667** | **0.22** | **0.238048** | **0** |
| TRIAL 2 | Methyl, Mito 5hmC, Nuc 5hmC, Mito 5mC, Nuc 5mC, CpG 1 - 24, TFAM CpG, TFAM Non-CpG | **0.541667** | **0.43** | **0.250693** | **0** |
| TRIAL 3 | Methyl, Mito 5hmC, Nuc 5hmC, Mito 5mC, Nuc 5mC, CpG 24, TFAM CpG, TFAM Non-CpG | **0.775** | **0.22** | **0.325** | **0** |
| TRIAL 4 | Methyl, Nuc 5mC, CpG 1 - 24 | **0.525** | **0.44** | **0.276512** | **0** |
| TRIAL 5 | Nuc 5mC, CpG 1,CpG 7,CpG 8,CpG 9,CpG 11,CpG 12,CpG 22,CpG 23,CpG 24,CpG 28 | **0.683333** | **0.78** | **0.306866** | **0** |
|  |  |  |  |  |  |
|  | **KNN Binary** | | | | |
|  | **Features Implemented in Training and Testing by Trial** | **Training (Average Accuracy)** | **Testing (F1 Score)** | **Train St.D Average** | **Test St. D** |
| TRIAL 1 | Methyl, Mito 5hmC, Nuc 5hmC, Mito 5mC, Nuc 5mC, CpG 11, CpG 23, CpG 24, CpG 28 | **0.608333** | **0.66** | **0.295921** | **0** |
| TRIAL 2 | Methyl, Mito 5hmC, Nuc 5hmC, Mito 5mC, Nuc 5mC, CpG 1 - 24, TFAM CpG, TFAM Non-CpG | **0.608333** | **0.78** | **0.229885** | **0** |
| TRIAL 3 | Methyl, Mito 5hmC, Nuc 5hmC, Mito 5mC, Nuc 5mC, CpG 24, TFAM CpG, TFAM Non-CpG | **0.716667** | **0.76** | **0.236291** | **0** |
| TRIAL 4 | Methyl, Nuc 5mC, CpG 1 - 24 | **0.633333** | **0.78** | **0.230338** | **0** |
| TRIAL 5 | Nuc 5mC, CpG 1,CpG 7,CpG 8,CpG 9,CpG 11,CpG 12,CpG 22,CpG 23,CpG 24,CpG 28 | **0.641667** | **0.66** | **0.258333** | **0** |
|  |  |  |  |  |  |
|  | **NB Binary** | | | | |
|  | **Features Implemented in Training and Testing by Trial** | **Training (Average Accuracy)** | **Testing (F1 Score)** | **Train St.D Average** | **Test St. D** |
| TRIAL 1 | Methyl, Mito 5hmC, Nuc 5hmC, Mito 5mC, Nuc 5mC, CpG 11, CpG 23, CpG 24, CpG 28 | **0.691667** | **0.33** | **0.300578** | **0** |
| TRIAL 2 | Methyl, Mito 5hmC, Nuc 5hmC, Mito 5mC, Nuc 5mC, CpG 1 - 24, TFAM CpG, TFAM Non-CpG | **0.566667** | **0.67** | **0.355121** | **0** |
| TRIAL 3 | Methyl, Mito 5hmC, Nuc 5hmC, Mito 5mC, Nuc 5mC, CpG 24, TFAM CpG, TFAM Non-CpG | **0.691667** | **0.54** | **0.255631** | **0** |
| TRIAL 4 | Methyl, Nuc 5mC, CpG 1 - 24 | **0.508333** | **0.77** | **0.366003** | **0** |
| TRIAL 5 | Nuc 5mC, CpG 1,CpG 7,CpG 8,CpG 9,CpG 11,CpG 12,CpG 22,CpG 23,CpG 24,CpG 28 | **0.6** | **0.56** | **0.357071** | **0** |
|  |  |  |  |  |  |
|  | **SVM Binary** | | | | |
|  | **Features Implemented in Training and Testing by Trial** | **Training (Average Accuracy)** | **Testing (F1 Score)** | **Train St.D Average** | **Test St. D** |
| TRIAL 1 | Methyl, Mito 5hmC, Nuc 5hmC, Mito 5mC, Nuc 5mC, CpG 11, CpG 23, CpG 24, CpG 28 | **0.625** | **0.66** | **0.321131** | **0** |
| TRIAL 2 | Methyl, Mito 5hmC, Nuc 5hmC, Mito 5mC, Nuc 5mC, CpG 1 - 24, TFAM CpG, TFAM Non-CpG | **0.591667** | **0.61** | **0.208999** | **0** |
| TRIAL 3 | Methyl, Mito 5hmC, Nuc 5hmC, Mito 5mC, Nuc 5mC, CpG 24, TFAM CpG, TFAM Non-CpG | **0.766667** | **0.61** | **0.238048** | **0** |
| TRIAL 4 | Methyl, Nuc 5mC, CpG 1 - 24 | **0.591667** | **0.61** | **0.208999** | **0** |
| TRIAL 5 | Nuc 5mC, CpG 1,CpG 7,CpG 8,CpG 9,CpG 11,CpG 12,CpG 22,CpG 23,CpG 24,CpG 28 | **0.708333** | **0.76** | **0.230489** | **0** |

**Table S9:** Machine learning analyses for all combined features using binary classification. CART = Classification and Regression Trees, LR = Logistic Regression, LDA = Linear Discriminant Analysis, KNN = K-Nearest Neighbors, NB = Naïve Bayes, SVM = Support Vector Machines. Nuc = Nuclear, Mito = Mitochondrial, 5mC = 5-methylcytosine, 5hmC = 5-hydroxymethylcytosine, Methyl = S-adenosyl methionine methyltransferase activity, CpG = cytosine nucleotide followed by a guanine nucleotide, TFAM CpG = total CpG methylation of the TFAM promoter region, TFAM Non-CpG = total methylation of the TFAM promoter region not at CpG sites, St.D. = standard deviation, binary = non-diabetic and type 2 diabetic, multiple = non-diabetic, prediabetic, and type 2 diabetic.

**Table S10**

| SEED = 86 | | | | | |
| --- | --- | --- | --- | --- | --- |
|  | **CART Multiple** | | | | |
|  | **Features Implemented in Training and Testing by Trial** | **Training (Average Accuracy)** | **Testing (F1 Score)** | **Train St.D Avg.** | **Test St. D** |
| TRIAL 1 | Mito 5mC, Nuc 5mC, Complex III, BMI, CpG 7, CpG 24, CpG 28, TFAM CpG, TFAM Non-CpG | **0.7916666** | **0.558** | **0.224768** | **0.00447214** |
| TRIAL 2 | Nuc 5mC, BMI, CpG 24, CpG 28, TFAM CpG, TFAM Non-CpG | **0.8016666** | **0.56** | **0.2271884** | **0** |
| TRIAL 3 | BMI, CpG 24, TFAM CpG, TFAM Non-CpG | **0.7316668** | **0.56** | **0.2568122** | **0** |
| TRIAL 4 | BMI, CpG 24, CpG 28, TFAM CpG, TFAM Non-CpG | **0.8366664** | **0.56** | **0.234217** | **0** |
| TRIAL 5 | BMI, CpG 24, TFAM CpG | **0.6933334** | **0.594** | **0.2764508** | **0.09476286** |
| TRIAL 6 | BMI, CpG 24, TFAM Non-CpG | **0.63** | **0.33** | **0.20036** | **0** |
| TRIAL 7 | BMI, CpG 24 | **0.6266668** | **0.258** | **0.2756018** | **0.0752994** |
|  |  |  |  |  |  |
|  | **LR Multiple** | | | | |
|  | **Features Implemented in Training and Testing by Trial** | **Training (Average Accuracy)** | **Testing (F1 Score)** | **Train St.D Avg.** | **Test St. D** |
| TRIAL 1 | Mito 5mC, Nuc 5mC, Complex III, BMI, CpG 7, CpG 24, CpG 28, TFAM CpG, TFAM Non-CpG | **0.516667** | **0.55** | **0.213437** | **0** |
| TRIAL 2 | Nuc 5mC, BMI, CpG 24, CpG 28, TFAM CpG, TFAM Non-CpG | **0.55** | **0.55** | **0.145297** | **0** |
| TRIAL 3 | BMI, CpG 24, TFAM CpG, TFAM Non-CpG | **0.516667** | **0.18** | **0.189297** | **0** |
| TRIAL 4 | BMI, CpG 24, CpG 28,TFAM CpG, TFAM Non-CpG | **0.516667** | **0.13** | **0.189297** | **0** |
| TRIAL 5 | BMI, CpG 24, TFAM CpG | **0.575** | **0.18** | **0.191667** | **0** |
| TRIAL 6 | BMI, CpG 24, TFAM Non-CpG | **0.558333** | **0.18** | **0.220637** | **0** |
| TRIAL 7 | BMI, CpG 24 | **0.533333** | **0.18** | **0.211476** | **0** |
|  |  |  |  |  |  |
|  | **LDA Multiple** | | | | |
|  | **Features Implemented in Training and Testing by Trial** | **Training (Average Accuracy)** | **Testing (F1 Score)** | **Train St.D Avg.** | **Test St. D** |
| TRIAL 1 | Mito 5mC, Nuc 5mC, Complex III, BMI, CpG 7, CpG 24, CpG 28, TFAM CpG, TFAM Non-CpG | **0.525** | **0.67** | **0.129368** | **0** |
| TRIAL 2 | Nuc 5mC, BMI, CpG 24, CpG 28, TFAM CpG, TFAM Non-CpG | **0.533333** | **0.43** | **0.15** | **0** |
| TRIAL 3 | BMI, CpG 24, TFAM CpG, TFAM Non-CpG | **0.475** | **0.44** | **0.232886** | **0** |
| TRIAL 4 | BMI, CpG 24, CpG 28,TFAM CpG, TFAM Non-CpG | **0.541667** | **0.44** | **0.125** | **0** |
| TRIAL 5 | BMI, CpG 24, TFAM CpG | **0.55** | **0.34** | **0.214735** | **0** |
| TRIAL 6 | BMI, CpG 24, TFAM Non-CpG | **0.475** | **0.19** | **0.232886** | **0** |
| TRIAL 7 | BMI, CpG 24 | **0.525** | **0.1** | **0.232886** | **0** |
|  |  |  |  |  |  |
|  | **KNN Multiple** | | | | |
|  | **Features Implemented in Training and Testing by Trial** | **Training (Average Accuracy)** | **Testing (F1 Score)** | **Train St.D Avg.** | **Test St. D** |
| TRIAL 1 | Mito 5mC, Nuc 5mC, Complex III, BMI, CpG 7, CpG 24, CpG 28, TFAM CpG, TFAM Non-CpG | **0.325** | **0.3** | **0.215542** | **0** |
| TRIAL 2 | Nuc 5mC, BMI, CpG 24, CpG 28, TFAM CpG, TFAM Non-CpG | **0.366667** | **0.33** | **0.284312** | **0** |
| TRIAL 3 | BMI, CpG 24, TFAM CpG, TFAM Non-CpG | **0.383333** | **0.31** | **0.26405** | **0** |
| TRIAL 4 | BMI, CpG 24, CpG 28,TFAM CpG, TFAM Non-CpG | **0.375** | **0.34** | **0.294038** | **0** |
| TRIAL 5 | BMI, CpG 24, TFAM CpG | **0.383333** | **0.31** | **0.286744** | **0** |
| TRIAL 6 | BMI, CpG 24, TFAM Non-CpG | **0.408333** | **0.31** | **0.26207** | **0** |
| TRIAL 7 | BMI, CpG 24 | **0.433333** | **0.13** | **0.23214** | **0** |
|  |  |  |  |  |  |
|  | **NB Multiple** | | | | |
|  | **Features Implemented in Training and Testing by Trial** | **Training (Average Accuracy)** | **Testing (F1 Score)** | **Train St.D Avg.** | **Test St. D** |
| TRIAL 1 | Mito 5mC, Nuc 5mC, Complex III, BMI, CpG 7, CpG 24, CpG 28, TFAM CpG, TFAM Non-CpG | **0.558333** | **0.4** | **0.16266** | **0** |
| TRIAL 2 | Nuc 5mC, BMI, CpG 24, CpG 28, TFAM CpG, TFAM Non-CpG | **0.608333** | **0.56** | **0.229885** | **0** |
| TRIAL 3 | BMI, CpG 24, TFAM CpG, TFAM Non-CpG | **0.608333** | **0.47** | **0.273988** | **0** |
| TRIAL 4 | BMI, CpG 24, CpG 28,TFAM CpG, TFAM Non-CpG | **0.591667** | **0.47** | **0.208999** | **0** |
| TRIAL 5 | BMI, CpG 24, TFAM CpG | **0.55** | **0.18** | **0.183333** | **0** |
| TRIAL 6 | BMI, CpG 24, TFAM Non-CpG | **0.566667** | **0.18** | **0.203443** | **0** |
| TRIAL 7 | BMI, CpG 24 | **0.55** | **0.18** | **0.214735** | **0** |
|  |  |  |  |  |  |
|  | **SVM Multiple** | | | | |
|  | **Features Implemented in Training and Testing by Trial** | **Training (Average Accuracy)** | **Testing (F1 Score)** | **Train St.D Avg.** | **Test St. D** |
| TRIAL 1 | Mito 5mC, Nuc 5mC, Complex III, BMI, CpG 7, CpG 24, CpG 28, TFAM CpG, TFAM Non-CpG | **0.35** | **0.17** | **0.152753** | **0** |
| TRIAL 2 | Nuc 5mC, BMI, CpG 24, CpG 28, TFAM CpG, TFAM Non-CpG | **0.491667** | **0.31** | **0.29451** | **0** |
| TRIAL 3 | BMI, CpG 24, TFAM CpG, TFAM Non-CpG | **0.408333** | **0.08** | **0.275** | **0** |
| TRIAL 4 | BMI, CpG 24, CpG 28,TFAM CpG, TFAM Non-CpG | **0.341667** | **0.13** | **0.29451** | **0** |
| TRIAL 5 | BMI, CpG 24, TFAM CpG | **0.383333** | **0.18** | **0.298608** | **0** |
| TRIAL 6 | BMI, CpG 24, TFAM Non-CpG | **0.491667** | **0.08** | **0.248468** | **0** |
| TRIAL 7 | BMI, CpG 24 | **0.466667** | **0.18** | **0.211476** | **0** |

**Table S10:** Machine learning analyses for all combined features using multiple classification. CART = Classification and Regression Trees, LR = Logistic Regression, LDA = Linear Discriminant Analysis, KNN = K-Nearest Neighbors, NB = Naïve Bayes, SVM = Support Vector Machines. Nuc = Nuclear, Mito = Mitochondrial, 5mC = 5-methylcytosine, 5hmC = 5-hydroxymethylcytosine, CpG = cytosine nucleotide followed by a guanine nucleotide, TFAM CpG = total CpG methylation of the TFAM promoter region, TFAM Non-CpG = total methylation of the TFAM promoter region not at CpG sites, St.D. = standard deviation, binary = non-diabetic and type 2 diabetic, multiple = non-diabetic, prediabetic, and type 2 diabetic.

**Table S11**

| **Model** | **Training** | **Training**  **(StDev)** | **Testing** | **Testing**  **(StDev)** | **F1 Score** | **Important Features** | **Important Feature Bias** | **AUC** |
| --- | --- | --- | --- | --- | --- | --- | --- | --- |
| **LR** | 0.808 | 0.171 | 0.667 | 0.000 | 0.670 | CpG 35, CpG 29, Mito 5mC, CpG 1, Nuc 5mC, 16362, SNP7028, CpG 24, SNP16519, SNP11719 | (2.656), (-1.496), (-0.699), (0.583), (0.535), (-0.359), (0.311), (-0.304), (0.099), (-0.066) | 0.700 |
| **LDA** | 0.917 | 0.129 | 0.778 | 0.000 | 0.780 | SNP4295, SNP4917, SNP7028, SNP16362, Nuc 5hmC, SNP16519, 72, Nuc 5mC, SNP8860, CpG 29 | (-1.050E+16), (-8.170), (7.367), (-6.313), (-3.659), (3.472), (3.113), (2.483), (-2.119), (-1.359) | 0.800 |
| **KNN** | 0.750 | 0.236 | 0.556 | 0.000 | 0.560 | NA | NA | 0.675 |
| **NB** | 0.683 | 0.249 | 0.778 | 0.000 | 0.780 | Mito 5hmC, Methyltransferase | (1.000), (0.000) | 0.725 |
| **SVM** | 0.750 | 0.242 | 0.667 | 0.000 | 0.670 | CpG 35, CpG 29, SNP16362, Mito 5mC, Nuc 5mC, SNP7028, 72, CpG 24, SNP16519, CpG 1 | (2.240), (-0.892), (-0.467), (-0.395), (0.313), (0.225), (0.216), (-0.210), (0.204), (0.190) | 0.650 |
| **CART** | 0.833 | 0.212 | 0.711 | 0.061 | 0.692 | CpG 24, Nuc 5mC, CpG 1 | (58.711%), (22.547%), (18.742%) | 0.740 |

**Table S11: Overview of 6 machine-learning model analysis on 18 selected features in binary classification.** Model analysis was conducted five times and averages are reported for the resulting training accuracy, training standard deviation, testing accuracy, testing standard deviation, F1 score, and *area under the curve* (AUC). Important biomarker features associated with each trained model are provided along with the associated influence value for each feature. Important features are listed in order of influence within the model. LR, LDA, SVM feature bias exists as an influence parameter where magnitude dictates feature influence. A positive influence value indicates the biomarker favors classification towards one label while a negative value indicates favorable classification of the opposite label. The larger the magnitude, the more strongly that feature shifts classification. NB feature influence indicates the most important biomarker per class in binary (0,1) classification schemes. CART feature bias percentages indicate feature influence on the created classification tree. Larger percentages indicate a feature that arises near the beginning of a tree before subsequent branching. Influence is not provided for KNN due to model restrictions. The 18 selected features include: Methyltransferase, Mito 5hmC, Nuc 5hmC, Mito 5mC, Nuc 5mC, CpG1, CpG24, CpG29, CpG35, SNP72, SNP4295, SNP4917, SNP7028, SNP8860, SNP11719, SNP16354, SNP16362, and SNP16519.

**Table S12**

| **Model** | **Training** | **Training**  **(StDev)** | **Testing** | **Testing**  **(StDev)** | **F1 Score** | **Important Features** | **Important Feature Bias** |
| --- | --- | --- | --- | --- | --- | --- | --- |
| **LR** | 0.408 | 0.209 | 0.444 | 0.000 | 0.380 | Nuc 5mC , CpG1, CpG29, SNP16362, SNP7028, CpG35, SNP16519, SNP11719, CpG24, Mito 5mC | (-0.381), (-0.244), (0.200), (0.191), (-0.168), (-0.122), (-0.099), (0.081), (0.047), (0.031) |
| **LDA** | 0.392 | 0.291 | 0.667 | 0.000 | 0.640 | CpG35, SNP16362, SNP7028, CpG29, SNP4917, SNP11719, Nuc 5mC, Nuc 5hmC, Methyltransferase, Mito 5mC | (-2.932), (1.759), (-1.431), (1.323), (1.232), (1.151), (-1.144), (0.899), (-0.767), (0.640) |
| **KNN** | 0.400 | 0.288 | 0.222 | 0.000 | 0.180 | NA | NA |
| **NB** | 0.367 | 0.312 | 0.444 | 0.000 | 0.350 | Nuc 5hmC, Mito 5hmC, Methyltransferase | (2.000), (1.000), (0.000) |
| **SVM** | 0.467 | 0.264 | 0.333 | 0.000 | 0.360 | CpG35, Nuc 5mC, Mito 5mC, CpG24, SNP7028, CpG29, SNP11719, CpG1, SNP4917, Mito 5hmC | (0.770), (0.645), (-0.560), (-0.348), (-0.195), (-0.165), (0.137), (0.028), (0.026), (0.011) |
| **CART** | 0.492 | 0.352 | 0.556 | 0.000 | 0.570 | CpG 24, Methyltransferase, CpG 29, Nuc 5mC, CpG 1, Mito 5hmC, Mito 5mC, Nuc 5hmC, SNP11719 | (32.762%), (21.973%), (19.800%), (8.501%), (5.598%), (3.357%), (2.975%), (1.678%), (1.678%) |

**Table S12: Overview of 6 machine-learning model analysis on 18 selected features in multiple classification.** Model analysis was conducted five times and averages are reported for the resulting training accuracy, training standard deviation, testing accuracy, testing standard deviation, and F1 score. Important biomarker features associated with each trained model are provided along with the associated influence value for each feature. Important features are listed in order of influence within the model. LR, LDA, SVM feature bias exists as an influence parameter where magnitude dictates feature influence. A positive influence value indicates the biomarker favors classification towards one label while a negative value indicates favorable classification of the opposite label. The larger the magnitude, the more strongly that feature shifts classification. NB feature influence indicates the most important biomarker per class in multiple (0,1,2) classification schemes. CART feature bias percentages indicate feature influence on the created classification tree. Larger percentages indicate a feature that arises near the beginning of a tree before subsequent branching. Influence is not provided for KNN due to model restrictions. The 18 selected features include: Methyltransferase, Mito 5hmC, Nuc 5hmC, Mito 5mC, Nuc 5mC, CpG1, CpG24, CpG29, CpG35, SNP72, SNP4295, SNP4917, SNP7028, SNP8860, SNP11719, SNP16354, SNP16362, and SNP16519.

**Table S13**

| **Parameter** | **Non-Diabetic** | **Type 2 Diabetic** |
| --- | --- | --- |
| Nuclear 5mC (% Methylation) | 2.039 ± 0.1063 | **2.873 ± 0.2445*** |
| Mitochondrial 5mC (% Methylation) | 1.776 ± 0.3107 | 1.644 ± 0.3658 |
| Nuclear 5hmC (% Methylation) | 0.178 ± 0.01935 | **0.1035 ± 0.0171*** |
| Mitochondrial 5hmC (% Methylation) | 0.08123 ± 0.02082 | 0.04837 ± 0.01984 |
| Methyltransferase (µmol/min/mL) | 0.005841 ± 0.0004433 | **0.004895 ± 0.0001608*** |
| Complex I (nmol/min/µg) | 45.24 ± 3.302 | **35.14 ± 3.193*** |
| Complex III (nmol/min/µg) | 106.8 ± 9.509 | **79.7 ± 6.337*** |
| Complex IV (nmol/min/µg) | 15.28 ± 1.7 | 16.52 ± 2.193 |
| Complex V (nmol/min/mg) | 134.7 ± 14.34 | 114.2 ± 10.1 |
| Citrate Synthase (Unit/mg mitochondria) | 0.02384 ± 0.00215 | 0.02021 ± 0.0009824 |
| TFAM CpG (% Methylation) | 1.663 ± 0.07412 | 1.568 ± 0.1121 |
| TFAM Non-CpG (% Methylation) | 1.267 ± 0.05839 | 1.237 ± 0.08055 |

**Table S13:** Biochemical features assessed in the non-diabetic and type 2 diabetic cohort. Groups are considered significantly different if *P* ≤ 0.05 = ***** compared to non-diabetic. All data are presented as the mean ± standard error of the mean (SEM). 5mC = 5-methylcytosine, 5hmC = 5-hydroxymethylcytosine, CpG = cytosine nucleotide followed by a guanine nucleotide, Complex = electron transport chain complex activity, TFAM = transcription factor A, mitochondrial.

**Table S14**

| **Parameter** | **Non-Diabetic** | **Pre-Diabetic** | **Type 2 Diabetic** |
| --- | --- | --- | --- |
| Nuclear 5mC (% Methylation) | 2.057 ± 0.1247 | 2.019 ± 0.1829 | **2.873 ± 0.2445*#** |
| Mitochondrial 5mC (% Methylation) | 1.448 ± 0.261 | 1.432 ± 0.2627 | 1.166 ± 0.191 |
| Nuclear 5hmC (% Methylation) | 0.1839 ± 0.02649 | 0.1617 ± 0.02873 | 0.107 ± 0.0169 |
| Mitochondrial 5hmC (% Methylation) | 0.07123 ± 0.03244 | 0.0964 ± 0.02049 | 0.05024 ± 0.02082 |
| Methyltransferase (µmol/min/mL) | 0.006339 ± 0.0006806 | 0.004878 ± 0.0004467 | 0.005185 ± 0.0002661 |
| Complex I (nmol/min/µg) | 42.69 ± 4.352 | 48.14 ± 5.091 | 35.14 ± 3.193 |
| Complex III (nmol/min/µg) | 105.2 ± 11.83 | 108.7 ± 15.72 | 79.7 ± 6.337 |
| Complex IV (nmol/min/µg) | 13.67 ± 1.738 | 17.12 ± 3.057 | 16.52 ± 2.193 |
| Complex V (nmol/min/mg) | 119.8 1 ± 3.07 | 151.7 ± 26.76 | 114.2 ± 10.1 |
| Citrate Synthase (Unit/mg mitochondria) | 0.01961 ± 0.001418 | 0.02868 ± 0.004009 | 0.02021 ± 0.0009824 |
| TFAM CpG (% Methylation) | 1.45 ± 0.05362 | **1.907 ± 0.1179*** | 1.568 ± 0.1121 |
| TFAM Non-CpG (% Methylation) | 1.2 ± 0.03096 | 1.343 ± 0.1192 | 1.237 ± 0.08055 |

**Table S14:** Biochemical features assessed in the non-diabetic and type 2 diabetic cohort. Groups are considered significantly different if *P* ≤ 0.05 = ***** compared to non-diabetic or **#** compared to pre-diabetic. All data are presented as the mean ± standard error of the mean (SEM). 5mC = 5-mehtylcytosine, 5hmC = 5-hydroxymethylcytosine, CpG = cytosine nucleotide followed by a guanine nucleotide, Complex = electron transport chain complex activity, TFAM = transcription factor A, mitochondrial.

**Figure S1**


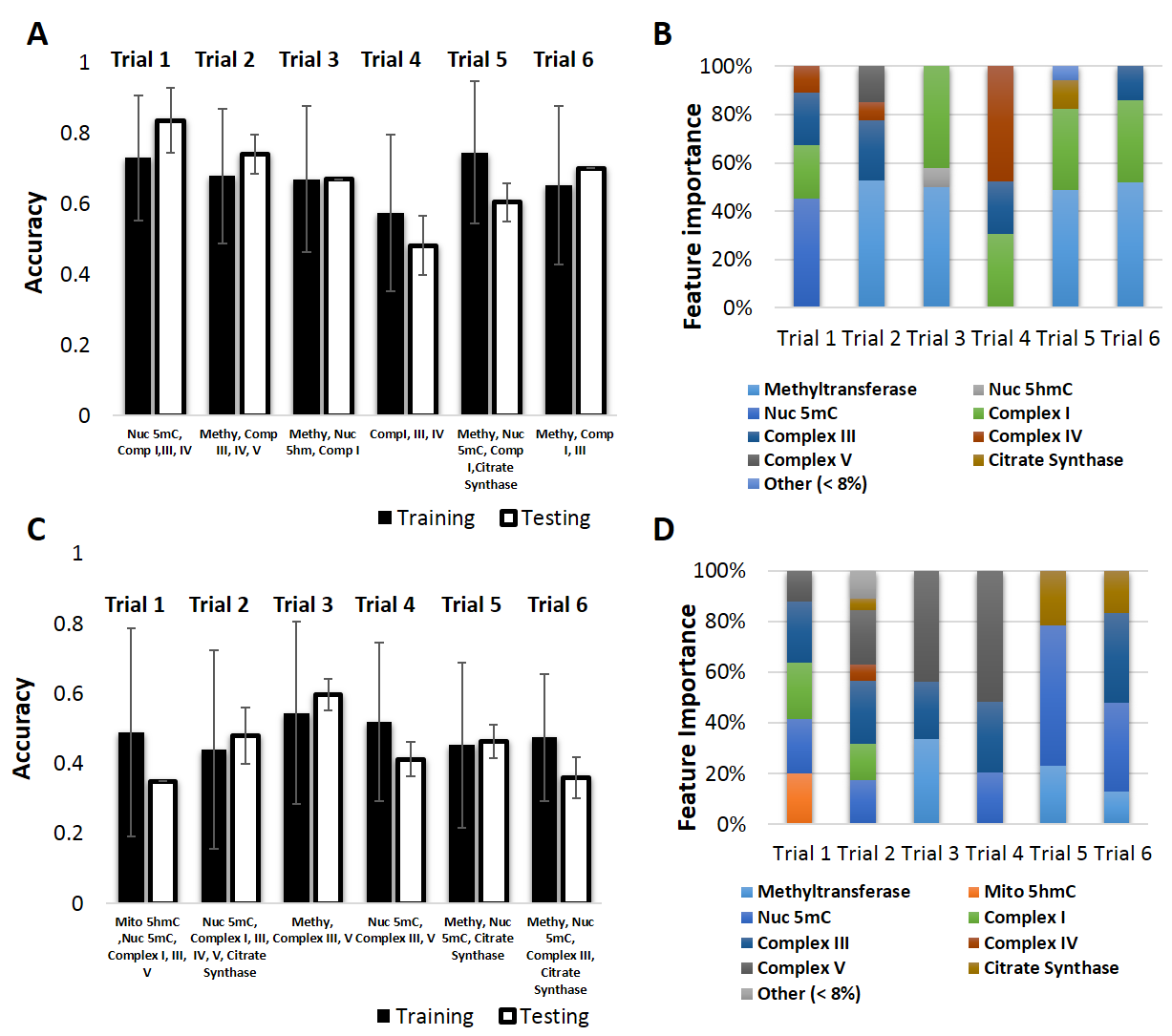


**Figure S1:** Feature importance using CART for physiological and biochemical characteristics from patients. (A) Training and testing accuracies for combinations of parameters that gave the most predictive outcomes in binary classification. (B) Percentage of individual feature importance by trial for binary classification. (C) Training and testing accuracies for combinations of parameters that gave the most predictive outcomes in multiple classification. (D) Percentage of individual feature importance by trial for multiple classification. Nuc = nuclear, Mito = mitochondrial, 5mC = 5-methylcytosine, 5hmC, 5-hydroxymethylcytosine, Methyl = S-adenosyl methionine methyltransferase activity, binary = non-diabetic and type 2 diabetic, multiple = non-diabetic, prediabetic, and type 2 diabetic.

**Figure S2**


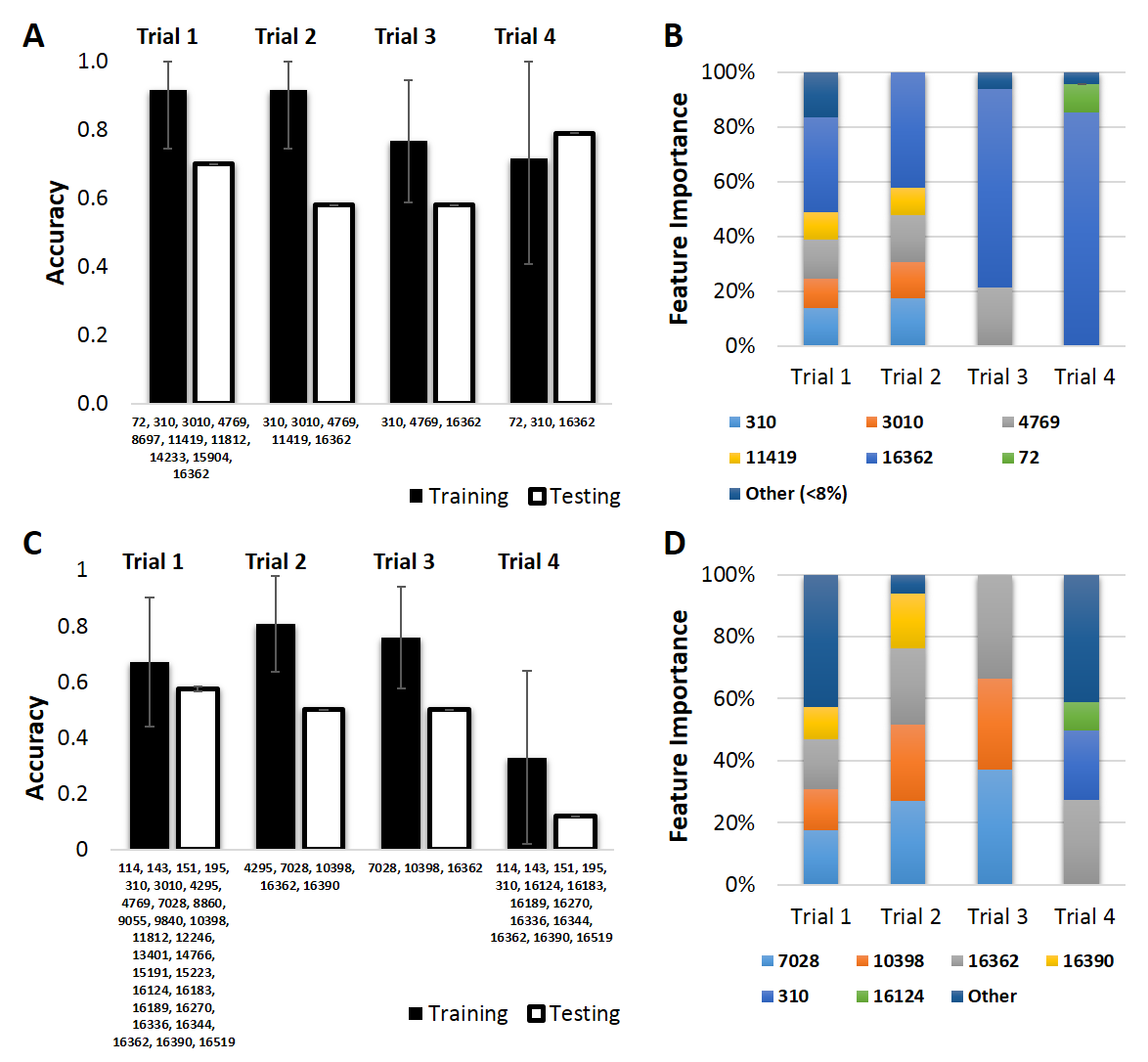


**Figure S2:** Feature importance using CART for mitochondrial DNA SNPs from patients. (A) Training and testing accuracies for combinations of parameters that gave the most predictive outcomes in binary classification. (B) Percentage of individual feature importance by trial for binary classification. (C) Training and testing accuracies for combinations of parameters that gave the most predictive outcomes in multiple classification. (D) Percentage of individual feature importance by trial for multiple classification. Numbers are indicative of the single nucleotide polymorphism (SNP) found in the mitochondrial DNA. Binary = non-diabetic and type 2 diabetic, multiple = non-diabetic, prediabetic, and type 2 diabetic.

**Figure S3**


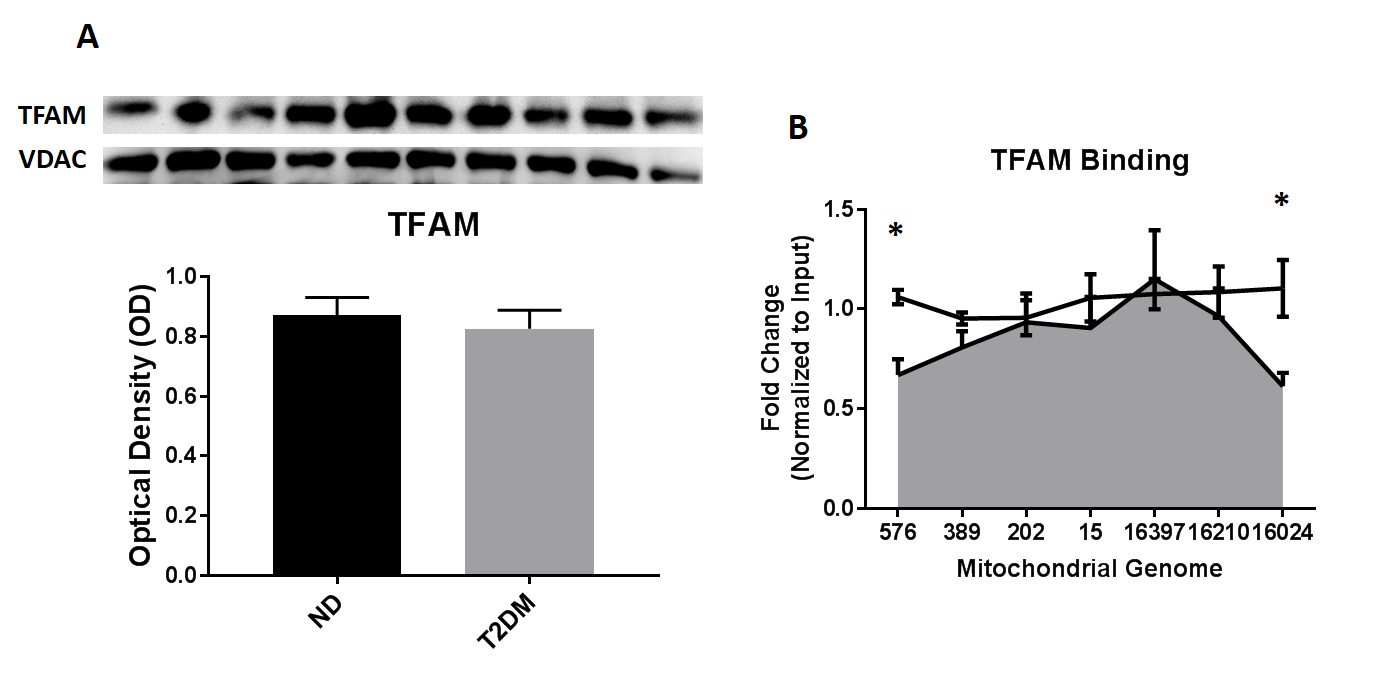


**Figure S3:** Expression and binding affinity of TFAM. (A) Expression of TFAM in isolated mitochondria from non-diabetic (n = 5, left) and type 2 diabetic (n = 5, right) patients. (B) Binding affinity of TFAM to the D-Loop region of mitochondrial DNA using chromatin immunoprecipitation. Groups are considered significantly different if *P* ≤ 0.05 = ***** compared to non-diabetic. All data are presented as the mean ± standard error of the mean (SEM). TFAM = transcription factor A, mitochondrial.

**Figure S4**


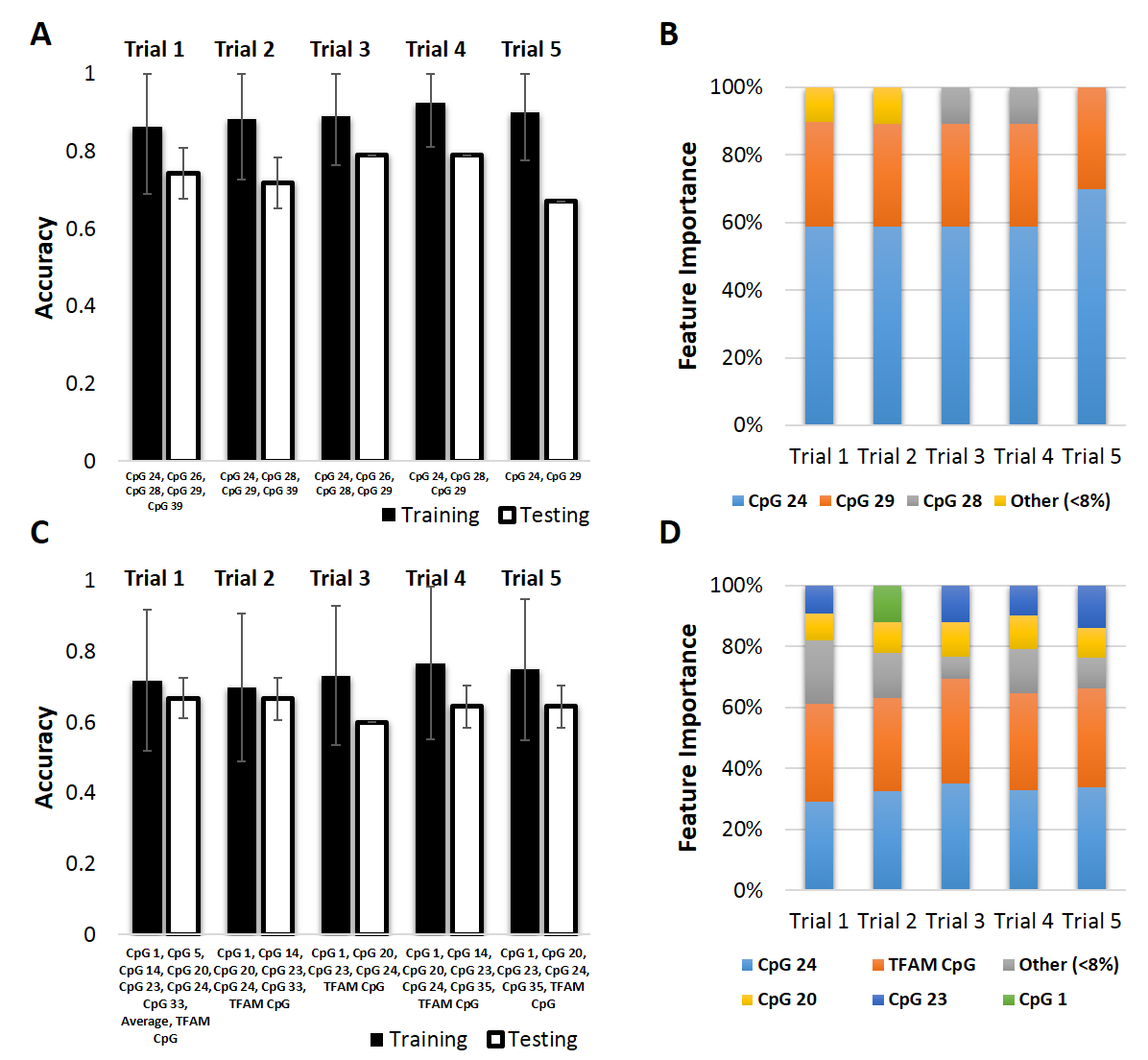


**Figure S4:** Feature importance using CART for CpG island methylation of TFAM from patients. (A) Training and testing accuracies for combinations of parameters that gave the most predictive outcomes in binary classification. (B) Percentage of individual feature importance by trial for binary classification. (C) Training and testing accuracies for combinations of parameters that gave the most predictive outcomes in multiple classification. (D) Percentage of individual feature importance by trial for multiple classification. CpG = cytosine nucleotide followed by a guanine nucleotide, TFAM CpG = total CpG methylation of the TFAM promoter region, TFAM = transcription factor A, mitochondrial, binary = non-diabetic and type 2 diabetic, multiple = non-diabetic, prediabetic, and type 2 diabetic.

**Figure S5**


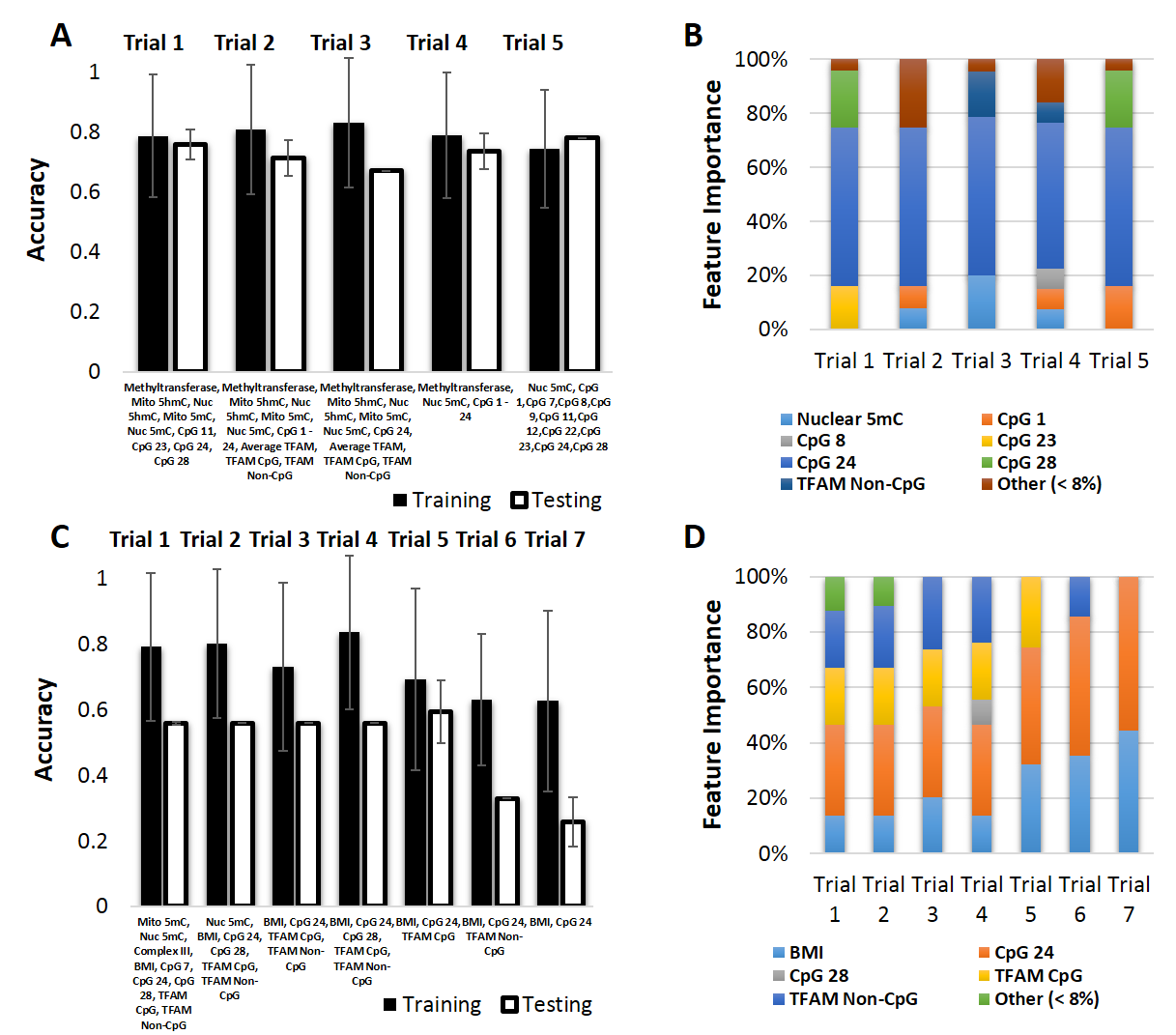


**Figure S5:** Feature importance using CART for all factors combined from patients. (A) Training and testing accuracies for combinations of parameters that gave the most predictive outcomes in binary classification. (B) Percentage of individual feature importance by trial for binary classification. (C) Training and testing accuracies for combinations of parameters that gave the most predictive outcomes in multiple classification. (D) Percentage of individual feature importance by trial for multiple classification. Nuc = nuclear, Mito = mitochondrial, 5mC = 5-methylcytosine, 5hmC, 5-hydroxymethylcytosine, Methylytransferase = S-adenosyl methionine methyltransferase activity, CpG = cytosine nucleotide followed by a guanine nucleotide, TFAM CpG = total CpG methylation of the TFAM promoter region, TFAM Non-CpG = total methylation of the TFAM promoter region not at CpG sites, TFAM = transcription factor A, mitochondrial, binary = non-diabetic and type 2 diabetic, multiple = non-diabetic, prediabetic, and type 2 diabetic.
